# Predictive analytics identifies key factors driving hyperalgesic priming of muscle sensory neurons

**DOI:** 10.3389/fnins.2023.1254154

**Published:** 2023-10-24

**Authors:** Sridevi Nagaraja, Shivendra G. Tewari, Jaques Reifman

**Affiliations:** ^1^Department of Defense Biotechnology High Performance Computing Software Applications Institute, Telemedicine and Advanced Technology Research Center, US Army Medical Research and Development Command, Fort Detrick, MD, United States; ^2^The Henry M. Jackson Foundation for the Advancement of Military Medicine, Inc., Bethesda, MD, United States

**Keywords:** musculoskeletal pain, nociceptor, ion channels, computational analysis, action potential, hyperalgesic priming, inflammation

## Abstract

Hyperalgesic priming, a form of neuroplasticity induced by inflammatory mediators, in peripheral nociceptors enhances the magnitude and duration of action potential (AP) firing to future inflammatory events and can potentially lead to pain chronification. The mechanisms underlying the development of hyperalgesic priming are not well understood, limiting the identification of novel therapeutic strategies to combat chronic pain. In this study, we used a computational model to identify key proteins whose modifications caused priming of muscle nociceptors and made them hyperexcitable to a subsequent inflammatory event. First, we extended a previously validated model of mouse muscle nociceptor sensitization to incorporate Epac-mediated interaction between two G protein-coupled receptor signaling pathways commonly activated by inflammatory mediators. Next, we calibrated and validated the model simulations of the nociceptor’s AP response to both innocuous and noxious levels of mechanical force after two subsequent inflammatory events using literature data. Then, by performing global sensitivity analyses that simulated thousands of nociceptor-priming scenarios, we identified five ion channels and two molecular processes (from the 18 modeled transmembrane proteins and 29 intracellular signaling components) as potential regulators of the increase in AP firing in response to mechanical forces. Finally, when we simulated specific neuroplastic modifications in Kv1.1 and Nav1.7 alone as well as with simultaneous modifications in Nav1.7, Nav1.8, TRPA1, and Kv7.2, we observed a considerable increase in the fold change in the number of triggered APs in primed nociceptors. These results suggest that altering the expression of Kv1.1 and Nav1.7 might regulate the neuronal hyperexcitability in primed mechanosensitive muscle nociceptors.

## Introduction

Acute pain arising from musculoskeletal injuries sometimes persists beyond the typical tissue-recovery time and transitions to chronic pain. The mechanisms underlying this transition from acute to chronic pain are not well understood, hampering the development of new treatment options (Heinricher, 2016; Pozek et al., 2016). However, we do know that primary afferent nociceptors in muscle tissue recognize noxious stimuli (e.g., heat and mechanical forces) and respond to them by firing action potentials (APs) via the action of numerous membrane proteins and intracellular molecules. These nociceptors can be sensitized by inflammatory mediators, such as prostaglandin E_2_ (PGE_2_), and develop long-lasting hyperexcitability (i.e., an increase in the magnitude and duration of AP firing) to a subsequent challenge, a phenomenon known as hyperalgesic priming (Gold and Flake, 2005; Reichling and Levine, 2009; Gold and Gebhart, 2010). Priming can occur at either the peripheral or central terminals (in the spinal cord) of nociceptors, and it is believed to initiate the transition from acute to chronic pain (Gold and Gebhart, 2010; Gangadharan and Kuner, 2013). Thus, the “primed” state represents a kind of “pain memory” that could potentially be reversed to treat chronic pain (Kandasamy and Price, 2015). However, the specific neuroplastic changes in the nociceptor that constitute this memory and maintain its priming are not well understood. We know that inflammatory mediators, such as PGE_2_ and tumor necrosis factor-α, activate a number of different signaling pathways within the neuron, including G protein-coupled receptors (GPCR), nuclear factor-κB, and mitogen-activated protein kinases (Niederberger and Geisslinger, 2008; Wuertz et al., 2012). However, only a few molecular mechanisms, such as protein kinase C (PKC) activity or PKC_*ε*_-dependent switching between the distinct GPCR pathways, are believed to lead to hyperalgesic priming (Reichling and Levine, 2009). Moreover, we still do not know the contribution of the different membrane proteins to the increased neuronal response in primed nociceptors. Because the AP response originates at the peripheral nociceptors before it is transmitted to other neurons, knowledge of the key neuroplastic modifications contributing to the priming of these nociceptors and of the AP responses to multiple inflammatory insults is necessary to improve our understanding of the pain transition process and to help identify potential therapeutic targets to prevent it. In addition, targeting the peripheral nervous system is advantageous because it provides an opportunity to avoid side effects from targeting neurons in the central nervous system, e.g., targeting opioid receptors present in spinal cord neurons to treat pain can paradoxically contribute to the development of priming in many different nociceptors (Khomula et al., 2019, 2021).

While numerous behavioral animal models of hyperalgesic priming have helped identify certain key molecules and pathways, including PKC activation by exchange protein directly activated by cAMP (Epac), among others (Aley and Levine, 1999; Hucho and Levine, 2007; Ferrari et al., 2013a; Araldi et al., 2016a,b), it is difficult to know whether targeting them can particularly regulate the responses of primary afferent nociceptors because pain behavior results from the aggregation of responses by many different neurons located in the peripheral and central nervous systems (Dubin and Patapoutian, 2010). On the other hand, *in vivo* models to assess primary afferent nociceptors, especially muscle afferents, are challenging because these neurons are heavily embedded in peripheral tissues (e.g., skin and muscle), making their nerve endings hard to access (Mense, 2010). In addition, quantifying the effects of priming through experiments involves blocking, knocking out, or enhancing individual ion channels or receptors in different inflammatory scenarios, some of which may not be feasible. Furthermore, these experiments are complicated by the presence of ongoing inflammation due to endogenous mediators, preventing the distinction between mechanisms of ongoing acute pain and the neuroplastic changes that specifically underlie the increase in the amount and frequency of AP firing and its prolongation during multiple inflammatory events.

Computational modeling can complement traditional experimentation in the search for key proteins or processes that could potentially regulate the response of primed nociceptors to future inflammatory events. Through computational modeling, we can compute the effects of various neuroplastic modifications, such as knocking out or overexpressing a given protein, or the effects of blocking or activating a molecular process on AP generation in response to a combination of different types of noxious stimuli and inflammatory mediators in a systematic and time-efficient manner. In fact, previous computational models of pain signaling in nociceptive neurons yielded insights into the roles of specific ion channels. For example, these models have characterized the contributions of different Na^+^ and K^+^ channels, such as TRPA1, Piezo2, Kv1.1, Nav1.6, Nav1.7, Nav1.8, and the Ca^2+^-activated K^+^ channel, to AP generation in neurons innervating the trigeminal nerve (Tanaka et al., 2016), gastrointestinal tract (Chambers et al., 2014), urinary bladder (Mandge and Manchanda, 2018), muscle tissue (Nagaraja et al., 2021), and other nonspecific afferent neuron dorsal root ganglions (Amir and Devor, 2003; Baker, 2005; Tigerholm et al., 2014). However, none of these analyses incorporated neuronal sensitization. The computational models that do account for inflammation-induced sensitization (Suleimanova et al., 2020), including the model recently developed by our group (Nagaraja et al., 2023), only represent one inflammatory event and do not account for the priming of muscle nociceptors and its effect on the AP response to subsequent inflammatory events. In addition, with one exception (Nagaraja et al., 2021, 2023), none of the previous models focused specifically on the signaling of muscle nociceptors. Given the high degree of variability exhibited by neurons depending on the physiological tissue they innervate, computational models must incorporate the specific membrane proteins known to be present on the pertinent neuron type. Therefore, to understand the mechanisms of hyperalgesic priming of muscle nociceptors and its effect on their responses to subsequent inflammatory events, we must use a computational model that represents membrane proteins and intracellular mechanisms specific to muscle nociceptors as well as the pathways activated within them by commonly encountered inflammatory mediators.

In this study, we primarily investigated the key transmembrane proteins and intracellular signaling processes that regulate the response of a mouse muscle nociceptor to mechanical forces during two subsequent inflammatory events. To this end, we extended a previously developed and validated mathematical model of inflammation-induced sensitization in a mechanosensitive muscle nociceptor (Nagaraja et al., 2023) to incorporate the kinetics of an Epac-mediated pathway activated by inflammatory mediators (i.e., PGE_2_) and a mechanosensitive membrane protein, TRPV4. The enhanced model represents 15 ion channels, two ion pumps, one ion exchanger, four endoplasmic reticulum (ER) membrane proteins, 29 intracellular components (including Ca^2+^ buffering proteins, kinases, enzymes, and second messenger molecules), and 46 associated processes. Upon model validation, we performed a global sensitivity analysis (GSA) by simulating the responses of 50,000 neurons to mechanical forces before and after the administration of an inflammatory mediator at two subsequent time points to quantify the contribution of the different modeled proteins and signaling processes to the change in AP firing magnitude after each inflammatory event. From this analysis, we first identified five ion channels and two processes as key regulators of the increase in neuronal AP firing in a primed muscle nociceptor (i.e., in the nociceptor after the second inflammatory event), and then investigated the effects of modifying these key regulators by introducing specific neuroplasticity after the first inflammatory event and evaluating the increase in the magnitude of neuronal AP firing after the second inflammatory event. These analyses allowed us to generate experimentally testable hypotheses regarding the role of ion channels and molecular processes in the priming of mouse muscle nociceptors and their response to mechanical stimuli.

## Materials and methods

### Computational model

**FIGURE 1:**
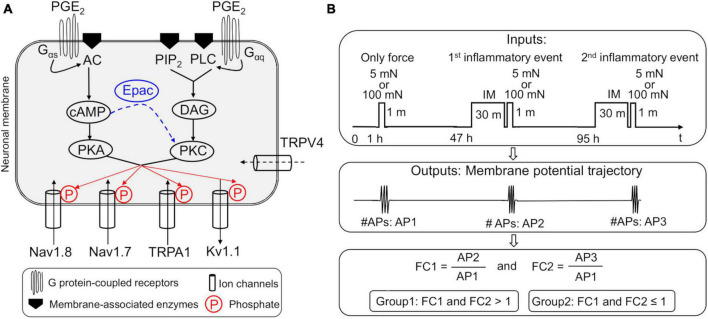
**(A)** Implementation of two parallel inflammation-induced G protein-coupled receptor (GPCR) signaling pathways in our nociceptive muscle neuron model. Shown are the four modeled neuronal transmembrane proteins, Nav1.8, Nav1.7, Kv1.1, and TRPA1, whose activation and inactivation kinetics are modified by protein kinases C (PKC) and A (PKA), which are activated by the two prostaglandin E_2_ (PGE_2_)-initiated intracellular signaling pathways in the model. The arrows of the ion channels indicate the direction of flow of the ions through the corresponding channel. In the first pathway, phosphorylation of the GPCR activates subunits G_αq_, β, and γ of the receptor. The G_αq_ subunit activates membrane-bound phospholipase C (PLC) and phosphatidylinositol 4,5-bisphosphate (PIP_2_) to produce diacylglycerol (DAG), which in turn activates PKC. In the second pathway, phosphorylation of the GPCR activates subunits G_αs_, β, and γ of the receptor. The G_αs_ subunit activates membrane-bound adenyl cyclase (AC) that activates cAMP, which in turn activates PKA. We also implemented the cAMP activation of Epac, which increases PKC activation (blue dashed arrow), thus providing feedback between the two inflammation-induced pathways. In addition, we added a mechanosensitive ion channel, TRPV4, previously not present in the model. Finally, we modeled the phosphorylation of Nav1.8, Nav1.7, Kv1.1, and TRPA1 by PKC and PKA, which modified their activation and inactivation kinetics. **(B)** Schematic showing the simulation inputs and outputs. In each simulation (one performed using the nominal parameter set and another using 50,000 distinct parameter sets generated for the global sensitivity analysis), we applied a step input of 5 or 100 mN mechanical force for 60 s at the simulation time point of 1 h. Next, at the 47- and 95-h simulation time points, we applied a step input of an inflammatory mediator (IM) at 100 nM for 30 min immediately followed by a 60-s step input of either 5 or 100 mN mechanical force. For each simulation that ran successfully, we used the time course of the membrane potential output to calculate the number of action potentials (APs) fired after the application of the mechanical force input once before (AP1) and twice after (AP2 and AP3) exposure to IM. Finally, we calculated the fold change in the number of APs fired after the first (FC1) and second (FC2) exposure to the IM, by dividing AP2 and AP3, respectively, by AP1. We classified the simulations where FC1 and FC2 were > 1 as primed neurons and those where FC1 and FC2 were ≤ 1 as non-primed neurons.

We extended a previously developed and validated model of inflammation-induced sensitization of muscle nociceptors to include intracellular interactions that contribute to hyperalgesic priming. The existing model simulates the effects of inflammatory mediators, such as PGE_2_, on neuronal responses via the independent activation of two distinct GPCR-mediated pathways, resulting in the activation of PKC and protein kinase A (PKA) within the neuron (Nagaraja et al., 2023). One of the GPCRs activates adenylyl cyclase, which catalyzes the activation of adenosine 3′,5′-cyclic monophosphate (cAMP). Under normal conditions, cAMP primarily activates PKA. However, after inflammation, cAMP also activates Epac, which in turn can activate PKC, thus providing feedback between the two GPCR-mediated signaling pathways (Hucho and Levine, 2007). To account for this feedback, we extended the model by adding descriptions of Epac kinetics (Figure 1A) and a new ion channel, i.e., TRPV4, based on evidence of its contribution to mechanical sensitivity in nociceptors (Ho et al., 2012; Mickle et al., 2015). The equations used to describe the Epac signaling and TRPV4 kinetics are provided in the Supplementary material.

### Model simulations, inputs, and outputs

Our extended model of inflammatory pain consists of 59 ordinary differential equations (ODEs) and 141 parameters. Each equation represents one variable in the model, where a variable represents activation or inactivation factors of 18 transmembrane proteins; the intracellular concentrations of K^+^, Na^+^, Ca^2+^, and inositol trisphosphate (IP_3_); the endoplasmic reticulum (ER) Ca^2+^ concentration; the active and inactive subunits of the two GPCRs; three membrane-associated enzymes; the concentrations of 18 intracellular proteins and second messenger molecules; and the membrane potential (*V*_*m*_). We provide a list of the model variables, their descriptions, and initial values in Supplementary Table 1. Using a lumped Hodgkin–Huxley-type formalism (Hodgkin and Huxley, 1952), we calculated changes in *V*_*m*_ at a given time point based on the changes in the currents of all neuronal transmembrane proteins listed above as follows:


(1) $$\frac{\text{d}\,V_{\text{m}}}{\text{d}\,t}=(I_{\mathrm{Nav1}\,.8}+I_{\mathrm{Nav1}\,.9}+I_{\mathrm{Nav1}\,.7}+I_{\text{Piezo}}+I_{\mathrm{ASIC3}}+I_{\text{TRPA}\,1}$$

$$+I_{\text{TRPV}\,4}+I_{\text{TREK}}+I_{\mathrm{Kv7}\,.2}+I_{\mathrm{Kv1}\,.1}+I_{\text{BKCa}}+I_{\text{Ka}}+I_{\text{Kleak}}$$

$$+I_{\text{CaT}}+I_{\text{CaL}}+I_{\text{PMCA}}+I_{\text{NaK}}+I_{\text{NCX}})/C{}_{\text{m}}$$

where *C*_*m*_ denotes the membrane capacitance and *I* represents the current through the different transmembrane proteins (described by the subscripts). The model simulates the time course of changes in the variables over 96 h in response to a mechanical force applied before and after two subsequent exposures to an inflammatory mediator (Figure 1B). We used 141 parameters to describe all the modeled mechanisms, and provide a list of the model parameter numbers (used to keep track of the parameters in our simulations), names, values, descriptions, units, and sources of the computational or experimental study from which we adapted or derived their values in Supplementary Table 2. We modified a subset of the model parameters (designated as “modified” in Supplementary Table 2) to match the inflammation-induced changes in AP firing magnitude from literature data (see “Model calibration and validation” section below). In all simulations, we maintained the extracellular concentrations of Na^+^, K^+^, and Ca^2+^ as well as the volume of the nociceptor nerve ending and its *C*_*m*_ at constant values (Nagaraja et al., 2023). We provide the ODEs and other equations describing all the modeled mechanisms, as well as the Nernst potentials and ionic balances for the intracellular concentrations of Na^+^, K^+^, and Ca^2+^ in the Supplementary material.

To drive the model, we provided as input a rectangular pulse with a mechanical force of either 5 or 100 mN (Figure 1B). We selected these values to represent inputs from both the innocuous and pain-inducing ranges of mechanical forces for mouse nociceptors (Cavanaugh et al., 2009; Deuis et al., 2017). We applied three pulses for a period of 60 s each, starting with the first pulse at the 1-h simulation time point. Next, at both the 47-h and 95-h simulation time points, we provided as input a 30-min rectangular pulse of an inflammatory mediator at a concentration of 100 nM, immediately followed by another 60-s rectangular pulse of 5 or 100 mN mechanical force. We chose the 30-min period to evaluate the inflammation-induced sensitization of the neuron’s response to mechanical forces based on literature data, which showed that the threshold reduction due to inflammation peaked between 30 min and 1 h in rat and mouse neurons (Hendrich et al., 2013). At the end of each simulation, our model generated a 96-h time course for each of the 59 model variables.

In all our computational analyses, we focused on the *V*_*m*_ time course, from which we calculated the total number of APs generated following the application of each pulse of mechanical force before and after the addition of the inflammatory mediator. We defined an AP as a *V*_*m*_ spike that crossed over 0 mV and reached a minimum value of +10 mV. We used the MATLAB function FINDPEAKS to identify the APs and to record their heights, widths, as well as the simulation time points at which they occurred. Next, we calculated two fold-change values (FC1 and FC2) for AP firing by dividing the total number of APs generated in response to the application of the force after the first and second inflammatory events (47 h and 95 h; Figure 1B), respectively, by the corresponding value before inflammation. The fold changes in the magnitude of AP firing following the two inflammatory events calculated using the nominal parameter set represented the baseline inflammation-induced sensitization. In addition to input forces of 5 and 100 mN, we calculated FC1 and FC2 values for force inputs of 10, 20, and 50 mN. We performed all computations in the software suite MATLAB R2015b (MathWorks, Natick, MA, USA) and solved the model equations using the MATLAB solver ODE15s with default tolerance levels.

### Model calibration and validation

#### Model calibration

To ensure that our model accurately captured the inflammation-induced increase in neuronal excitability (i.e., AP firing) as measured experimentally, we calibrated a subset of 10 out of the 141 model parameters using experimental data from the literature (Bar et al., 2004). To perform the calibration, we modified the values of 10 parameters associated with Epac and TRPV4 activation and inactivation, and Epac-mediated PKC activation (designated as “modified” in Supplementary Table 2), such that the simulated fold changes in the magnitude of AP firing to mechanical forces of 20 and 100 mN after two subsequent exposures to the inflammatory mediator were within ± 1.96 standard error of the corresponding experimental measurements in rat spinal neurons (Bar et al., 2004). We defined the model’s “nominal parameter set” as the final parameter values obtained after performing the calibration procedure.

#### Model validation

To validate our model, we compared its predictions (using the nominal parameter set) with literature data not used for model calibration. First, we compared the predictions of fold change in the number of APs fired in response to each of two mechanical forces of 100 mN after an exposure to 100 nM PGE_2_ at two subsequent time points with the corresponding experimental data from rat neurons (Hendrich et al., 2012). Second, using experimental data from the same study, we compared the predictions of the reduction in mechanical threshold (i.e., the minimum amount of force needed to elicit an AP) following the addition of 100 nM PGE_2_ at the two time points.

### Sensitivity analysis

We performed a sensitivity analysis to identify proteins and intracellular molecules and their associated signaling processes whose alterations were key for the development of hyperalgesic priming in the neuron and regulation of subsequent inflammation-induced changes in neuronal AP firing. First, we assessed the model’s robustness by performing a local sensitivity analysis (LSA), where we varied the model parameters near their nominal values (± 1%) as previously described (Nagaraja et al., 2014). Second, we performed a global sensitivity analysis (GSA) to account for the known heterogeneity in the expression of the various proteins at different nerve endings of muscle nociceptors. This analysis allowed us to account for the known variability in the conductance, activation, and inactivation gating factors of the same membrane proteins under different stimuli (Gold and Gebhart, 2010).

To this end, we simulated 50,000 distinct nociceptors and stimulated them with two inflammatory events followed by a mechanical force. First, we generated 50,000 unique parameter sets by randomly selecting parameter values from a fourfold range (twofold in each direction) around the nominal parameter values. To generate the random parameter sets, we used Latin hypercube sampling (MATLAB function LHSDESIGN) (Nagaraja et al., 2014). Next, we performed two sets of simulations using the 50,000 parameter sets, where we drove each simulation using a mechanical force of either 5 or 100 mN applied once before and twice 30 min after the addition of an inflammatory mediator at the 47- and 95-h simulation time points (Figure 1B). We interrupted and eliminated the time course simulations of *V*_*m*_ that did not reach the 96-h time point within 15 min of computation time (wall-clock) or that required time steps smaller than 1 × 10^–12^ s. We used this lack of convergence in the simulations to flag parameter sets that resulted in non-physiological kinetic behavior. Accordingly, we only used the simulations that ran to completion to calculate the fold changes FC1 and FC2 described above in the number of APs fired (in response to either 5 or 100 mN force) after each of the two inflammatory events. We used the calculations of APs, FC1, and FC2 to further eliminate the simulations in each force group where the number of APs fired in response to a mechanical force in the absence or presence of an inflammatory mediator was zero. Finally, we eliminated the simulations in which the AP fold-change values, FC1 and FC2, after each of the two inflammatory events were equal to one. We assumed that these simulations represent “non-nociceptive neurons” because their responses to noxious mechanical forces did not change after two consecutive inflammatory events, i.e., they did not have the ability to sensitize. Using the results from the remaining simulations, we performed two analyses, a partial rank correlation coefficient analysis and a parameter distribution analysis.

#### Partial rank correlation coefficient analysis

For this analysis, we calculated the Spearman’s partial rank correlation coefficient (PRCC) and the associated *p*-values between the primary outputs (i.e., the AP firing fold changes after each inflammatory event, FC1 and FC2) and each of the 141 model parameter values. The values of the PRCC varied between −1 and +1, with large absolute values reflecting a high impact of the model parameter on the model output (i.e., FC1 and FC2). The sign of the PRCC indicated the positive or negative directionality of the correlation between the model parameter and the output. A PRCC with a *p*-value < 0.01 indicated that it was significantly different from zero. Upon completion of this analysis, we obtained two sets of 141 PRCC values for each force input (i.e., 5 and 100 mN), along with their associated *p*-values.

#### Parameter distribution analysis

For this analysis, we first separated the simulations for each of the two input forces of 5 and 100 mN into two groups. We defined a group of simulations in which both AP fold changes FC1 and FC2 were > 1 as “primed” neurons and a group in which both FC1 and FC2 were ≤ 1 as “non-primed” neurons. Next, we generated histograms of the parameter value distributions for each of the model’s 141 parameters using the MATLAB function HIST, with 50 bins partitioning the interval between the minimal and maximal values for each model parameter in the two groups of simulations (i.e., primed and non-primed neurons). We calculated the percentage of the simulations for each distribution curve by dividing the number of simulations in which a given parameter’s value fell within the range of a bin by the total number of simulations in that group. Then, for each model parameter, we quantified the area of overlap between the sensitized and non-sensitized neuron group distributions by calculating the Bhattacharyya coefficient, which varied between 0 and 1, representing no and 100% overlap, respectively, as previously described (Mitrophanov et al., 2015). A small overlap area indicated that a parameter (and the protein it represents) was consistently over (or under) expressed in a primed neuron relative to a non-primed neuron and was therefore more likely to be associated with inflammation-induced neuronal hyperalgesic priming than parameters with larger distribution overlap areas.

We utilized the results from the PRCC and the parameter distribution analyses to identify key transmembrane proteins whose alterations could regulate the hyperalgesic priming in muscle nociceptors after inflammation. Using the results from the PRCC analysis, for each force input, we divided the set of 141 PRCCs into five clusters using a k-means clustering algorithm (MATLAB function KMEANS) (Nagaraja et al., 2017). We considered the model parameters in the cluster that had the highest absolute PRCC values and had *p*-values ≤ 0.01 as key contributors to hyperalgesic priming induced after inflammation. Using the results from the parameter distribution analysis, we first ranked the absolute values of the 141 Bhattacharyya coefficients in ascending order and designated the parameters within the top five lowest values as key for hyperalgesic priming of the neuron. Finally, we combined the model parameters identified as key in both analyses and labeled the proteins/molecules or the intracellular signaling processes represented by those parameters as key for AP-response regulation during multiple inflammatory events.

### Ion channel modification simulations

For each transmembrane protein identified as key for FC1 and FC2 changes, we performed simulations to mimic specific modifications associated with that protein which are likely to occur following the first inflammatory event that “primes” its response to future inflammatory events. In these simulations, we either reduced or increased the expression of a transmembrane protein at 48 h into the simulation (i.e., after the first inflammatory event). To simulate an increase or decrease in a protein’s expression, we multiplied or divided, respectively, the current in Eq. (1) corresponding to that protein by a factor of 2. First, we simulated the protein modifications described above using a model with the nominal parameter set, which represented an average nociceptive muscle afferent neuron. Then, to verify that we could reproduce the effects of different modifications in a population of neurons, we repeated the simulations for every modification using parameter sets from simulations that were classified as primed and non-primed neurons. As in the GSA, we interrupted the simulations that did not reach the 96-h time point of the *V*_*m*_ time course within 15 min of computation time (wall-clock) to flag parameter sets where a modification resulted in non-physiological kinetic behavior. Of the simulations that converged successfully, we calculated the mean ± 1 standard error (SE) of the AP fold changes (FC1 and FC2) after each inflammatory event for every modification. Finally, we performed a Wilcoxon rank sum test to compare the mean values of each simulation with a modification to the corresponding simulation without any modification.

## Results

### The model captured inflammation-induced changes in the activation threshold and in the AP firing response to mechanical stimulation

**FIGURE 2:**
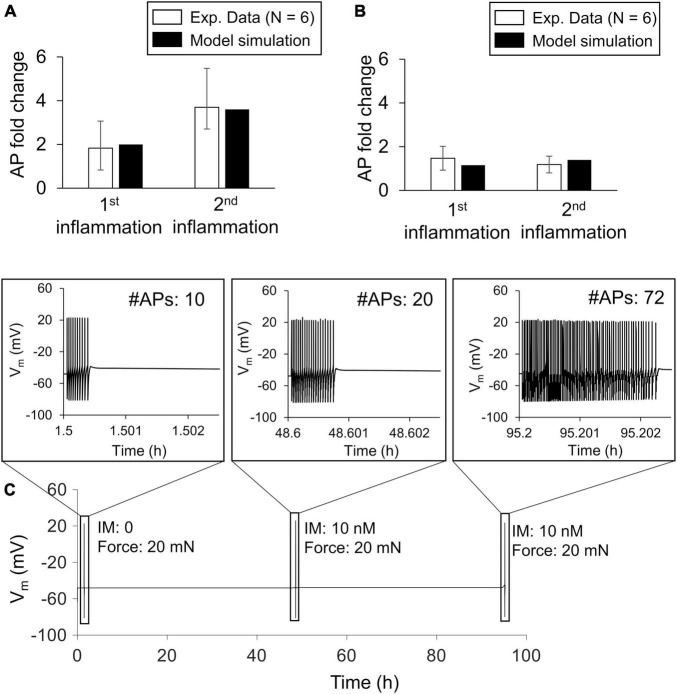
Model calibration. We calibrated the model’s predictions of the increase in action potential (AP) firing magnitude by fitting them to experimental data from rat spinal cord neurons in response to mechanical forces applied after the addition of an inflammatory mediator. Open bars (*N* = 6) show mean experimental data ± 1 standard error fold change in the magnitude of AP firing in response to innocuous **(A)** and noxious forces **(B)** after two subsequent exposures to inflammatory mediators (Bar et al., 2004). Solid bars show the results of model fitting to the experimental data. We used innocuous and noxious forces of 20 and 100 mN, respectively, to stimulate the neuron in the model. **(C)** Simulated time course trajectory of the membrane potential output in response to a 20 mN force applied before (at the 1-h time point) and after two subsequent inflammatory events (at the 47- and 95-h time points). The inset shows the shape and number of APs generated in each response.

To ensure that the model accurately captured the effect of multiple inflammatory events on the magnitude of the AP firing response to mechanical forces, we calibrated the model using data obtained from electrophysiological measurements in rat spinal neurons with peripheral terminals in the knee joint (Bar et al., 2004). The calibration procedure resulted in fold changes in the number of APs fired by the neuron in response to an innocuous (i.e., 20 mN; Figure 2A) and a noxious (100 mN; Figure 2B) mechanical force input after two subsequent applications of inflammatory mediators to fall within one SE of the experimental data. We designated the final set of model parameter values obtained after this calibration procedure as the nominal parameter set. Figure 2C shows the simulated APs for the case where we stimulated the neuron by a 20 mN force before (i.e., at the 1-h time point) and at 30 min after the addition of PGE_2_ at the 47- and 95-h simulation time points. In addition, based on the results of the LSA (performed using the nominal parameter set), we found that *V*_*m*_ was not overly sensitive (sensitivity indices > 100) to any of the model’s 141 parameters, suggesting that the model was stable and robust to small perturbations (± 1%) of its nominal values.

**FIGURE 3:**
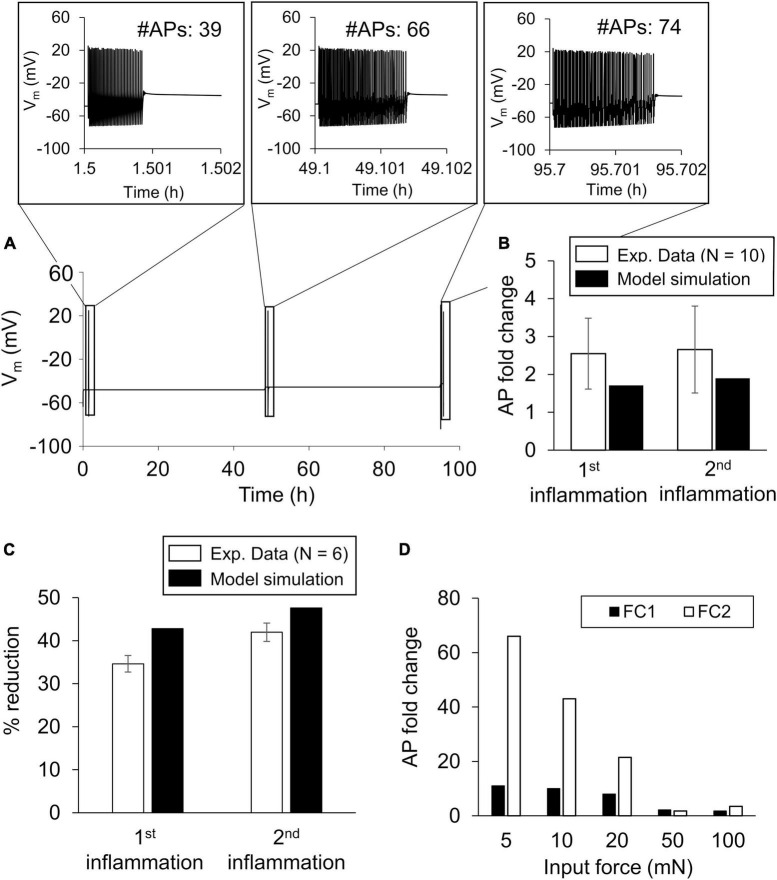
Model validation. We validated the model by comparing the simulations of inflammation-induced action potential (AP) firing increase and mechanical threshold reduction with the corresponding experimental data. **(A)** Simulated time course trajectory of the membrane potential output in response to a 100 mN force applied before (at the 1-h time point) and after two subsequent inflammatory events (i.e., at the 47- and 95-h time points). The inset shows the number of APs generated and their magnitude. **(B)** Mean experimental data ± 1 standard error (SE) fold change in the number of APs fired in response to a noxious force after exposure to two subsequent inflammatory events in rat gastrocnemius muscle neurons (open bars, *N* = 10) (Hendrich et al., 2013). **(C)** Mean experimental data of the percentage reduction ± 1 SE in the mechanical threshold induced by two subsequent exposures to 100 nM PGE_2_ (open bars, *N* = 6). Solid bars in **(B,C)** show the corresponding model predictions. **(D)** Model predictions of the AP firing fold change in response to five different force stimulations after two inflammatory events. The fold changes after the first inflammatory event are referred to as FC1 and those after the second inflammatory event as FC2.

To validate the model, we simulated the AP response (based on the nominal parameter set) to a mechanical force of 100 mN applied before (i.e., at the 1-h time point) and at 30 min after the administration of 100 nM PGE_2_ at the 47- and 95-h simulation time points and determined the number of APs fired after each stimulus (Figure 3A). We first compared the model predictions of the fold changes (FC1 and FC2) with the corresponding experimental data derived from rat gastrocnemius muscle neurons (Figure 3B). In addition, we compared the model predictions of the percentage reduction in the mechanical threshold (i.e., the minimum force required to elicit an AP from a neuron) 30 min after the addition of 100 nM PGE_2_ at the two time points in the simulation with the corresponding data in rat gastrocnemius muscle neurons (Hendrich et al., 2013). In the model, before the addition of the inflammatory mediator, the minimum force needed to elicit an AP was 1 mN. In both comparisons, our predictions were within ± 1.96 SE of the validation experimental data for all the comparisons, indicating that we cannot differentiate between the average results of the experiment and the model predictions (Figure 3C, error bars show 1 SE of experimental data).

Upon validation, we used the model to establish the baseline inflammation-induced increase in AP firing after two inflammatory events. The AP fold-change values for the individual forces after the first inflammatory event (FC1) were 11 for 5 mN, 10 for 10 mN, 8 for 20 mN, 2.1 for 50 mN, and 1.8 for 100 mN (Figure 3D, solid bars). The AP fold-change values for the individual forces after the second inflammatory event (FC2) were 66 for 5 mN, 43 for 10 mN, 21 for 20 mN, 1.75 for 50 mN, and 3.5 for 100 mN (Figure 3D, open bars). The fold-change values after either inflammatory event decreased as the intensity of the mechanical force stimulus increased.

**FIGURE 4:**
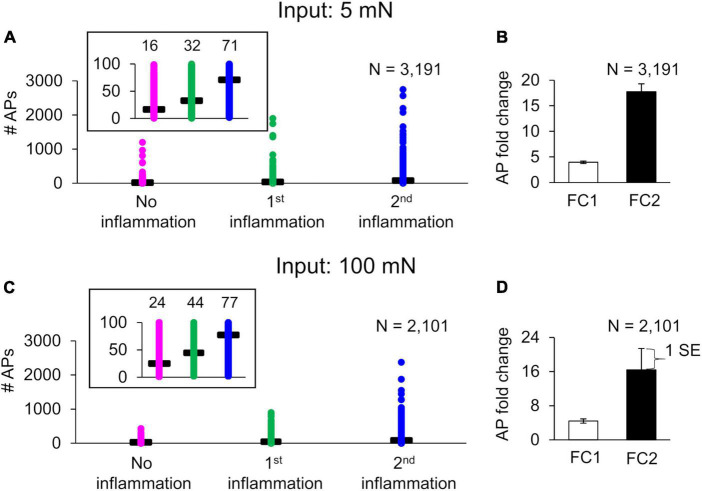
Baseline inflammation-induced sensitization and hyperalgesic priming of muscle nociceptors. Shown are the number of action potentials (APs) fired in response to a mechanical force of **(A)** 5 mN and **(C)** 100 mN applied before (pink circles) and after the first (green circles) and second (blue circles) exposures to an inflammatory mediator. The insets show the mean (horizontal black lines) number of APs fired. **(B,D)** show the corresponding mean ± 1 standard error (SE) of the AP fold-change values after the first (FC1) and second (FC2) inflammatory events in response to 5 and 100 mN mechanical forces, respectively.

For the next set of simulations, we selected 5 and 100 mN to represent a mechanical force input from the innocuous and pain-inducing range, respectively, for mouse nociceptors. In addition to simulating two inflammatory events using the nominal parameter set, we repeated the simulations using 50,000 virtual neurons. Of the 50,000 simulations performed for both the 5 and the 100 mN mechanical force inputs, 48,588 and 48,651, respectively, ran successfully. After two additional filtering procedures (see section “Sensitivity analysis” in “Materials and methods”), we extracted 4,310 and 4,027 simulations from the 5 and 100 mN groups, respectively, to use for further analysis. Lastly, in both groups, we classified the simulations as either “primed” or “non-primed” neurons. For the 5 mN simulation set, we identified 3,191 and 1,119 simulations as primed and non-primed neurons, respectively, and for the 100 mN simulation set, we identified 2,101 and 1,926 simulations as primed and non-primed neurons, respectively. Figures 4A, C shows the number of APs fired before (pink circles) and after the first (green circles) and second (blue circles) inflammatory events for the primed neurons in both the 5 and 100 mN groups, where the insets show the average APs fired (horizontal black bars). For both the 5 and 100 mN force inputs, the average fold changes in the number of APs fired in response to the second inflammatory event compared to those fired in the absence of inflammation (FC2; Figures 4B, D, solid bars) were higher than those for the first inflammatory event (FC1; Figures 4B, D, open bars). Overall, these results show that our simulations were able to generate a population of neurons in which hyperalgesic priming occurs in response to both innocuous and noxious stimuli.

### Key proteins and processes of inflammation-induced AP response regulation

**FIGURE 5:**
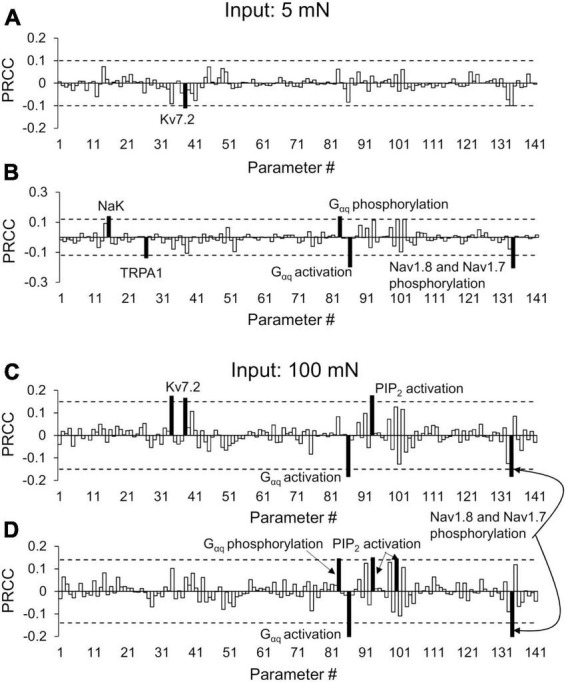
Partial rank correlation coefficient (PRCC) analysis identified key proteins and processes for action potential (AP) regulation. The bars show the PRCCs of the 141 model parameters with fold changes in the total number of APs generated after the first (FC1) and second (FC2) inflammatory events in response to a mechanical force of 5 mN **(A,B)** and 100 mN **(C,D)** in the primed neuron simulations. The PRCCs above their respective thresholds (dotted horizontal lines) that were statistically significant (i.e., *p* < 0.01; *p*-values depict the probability of seeing the observed correlation if no correlation exists) are indicated by solid black bars, and the labels of the bars show the ion channels/ion pumps or the rates of intracellular processes that these parameters describe in the model. We used the Spearman’s rank correlation method to compute the PRCC values, and obtained the *p*-values of the correlation using a Student’s *t*-test. Because the data were not normally distributed, we used a large-sample approximation while performing this test. The analyses are based on 2,438 and 1,605 simulations classified as primed neurons for applied mechanical forces of 5 and 100 mN, respectively.

To identify the transmembrane proteins that strongly regulated the AP response (specifically the number of APs generated) following the addition of an inflammatory mediator to a pre-inflamed muscle nociceptor across many different nociceptor-signaling conditions, we used two distinct analyses (PRCC and parameter distribution; see section “Sensitivity analysis” in “Materials and methods”). We used the AP fold-change values (FC1 and FC2) and the respective parameter values for each simulation group to perform the PRCC and parameter distribution analyses. For the simulations in which we used a mechanical force of 5 mN as input, the PRCC analysis results showed that for the primed neurons, the model parameters associated with Kv7.2 yielded high and statistically significant correlations (*p* < 0.01) with the FC1 values (Figure 5A, solid black bar), and the model parameters associated with proteins NaK and TRPA1 as well as molecular processes of G_α*q*_-coupled receptor phosphorylation and phosphorylation of Nav1.8 and Nav1.7 yielded high and statistically significant correlations (*p* < 0.01) with the FC2 values (Figure 5B, solid black bars). Moreover, for the non-primed neuron simulations, the PRCC analysis results showed that the model parameters associated with proteins NaK, Piezo2, and Nav1.7 yielded high and statistically significant correlations (*p* < 0.01) with the FC1 values (Supplementary Figure 1A, solid black bars), and the model parameters associated with proteins TRPA1 and Nav1.7 and G_α*q*_-coupled receptor phosphorylation yielded high and statistically significant correlations (*p* < 0.01) with the FC2 values (Supplementary Figure 1B, solid black bars).

For the simulations in which we used a mechanical force of 100 mN as input, the PRCC analysis results showed that the model parameters associated with Kv7.2 yielded high and statistically significant correlations (*p* < 0.01) with the FC1 values (Figure 5C, solid black bars), and the molecular processes of G_α*q*_-coupled receptor phosphorylation and activation, phosphatidylinositol 4,5-bisphosphate (PIP_2_) activation, and Nav1.8, and Nav1.7 phosphorylation by PKA and PKC yielded high and statistically significant correlations (*p* < 0.01) with both the FC1 and FC2 values (Figures 5C, D, solid black bars). For the non-primed neuron simulations, the PRCC analysis results showed that the model parameters associated with TRPA1 yielded high and statistically significant correlations (*p* < 0.01) with the FC1 values (Supplementary Figure 1C, solid black bar), and the model parameters associated with the activation of proteins Nav1.8 and Nav1.7 yielded high and statistically significant correlations (*p* < 0.01) with the FC2 values (Supplementary Figure 1D, solid black bars).

**FIGURE 6:**
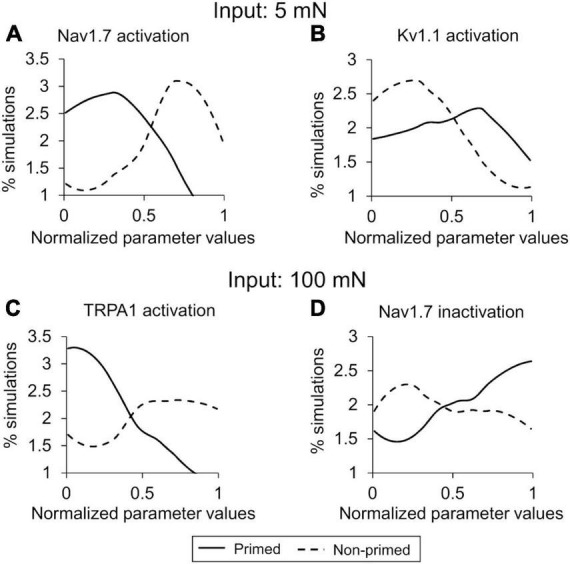
Parameter distribution analysis identified key proteins and processes for action potential (AP) regulation. Shown are the distributions of parameter values across the simulations of primed (solid lines) and non-primed (dashed lines) neuron groups representing **(A)** Nav1.7 activation and **(B)** Kv1.1 activation with a 5 mN mechanical force used as input, and **(C)** TRPA1 activation and **(D)** Nav1.7 inactivation with a 100 mN mechanical force used as input. The *x*-axis indicates the normalized parameter values, and the *y*-axis represents the percentage of simulations in each neuron group in which the parameter values fell within a particular range (described in the “Materials and methods” section).

In the parameter distribution analyses, we calculated the 141 Bhattacharyya coefficients to determine the overlap between the distributions of the normalized values of the model’s 141 parameters in the primed and non-primed neuron groups for both the 5 and 100 mN simulations. While none of the parameters had a considerably small overlap area between the two groups of 141 parameters, the parameters that demonstrated the five lowest values in the 5 mN group were associated with activation or inactivation of ion channels Nav1.7, Kv1.1, and TRPA1 and the rates of Nav1.8 and Nav1.7 phosphorylation by PKC and PKA post-inflammation. In the 100 mN simulation group, the parameters that demonstrated the five lowest values were associated with activation or inactivation of ion channels TRPA1, TRPV4, and Nav1.7 and the rates of TRPA1 phosphorylation by PKC and PKA post-inflammation. Supplementary Tables 3, 4 provide a list of the 141 Bhattacharyya coefficients for all model parameters for the 5 and 100 mN simulation groups, respectively. Figure 6 shows representative examples of two such parameters from each simulation set. For the parameters representing Nav1.7 activation (in the 5 mN force simulation group) and TRPA1 activation (in the 100 mN force simulation group), a larger percentage of the simulations in the primed neuron group fell in the lower range of their normalized values compared to those of the non-primed group (Figures 6A, C, solid vs. dashed lines), indicating that Nav1.7 and TRPA1 activation might be downregulated in primed neurons. Conversely, for the parameters representing Kv1.1 activation (in the 5 mN force simulation group) and Nav1.7 inactivation (in the 100 mN force simulation group), a larger percentage of the simulations in the primed neuron group fell in the higher range of their normalized values compared to those in the non-primed neuron group (Figures 6B, D, solid vs. dashed lines), indicating that these processes might be upregulated in primed neurons. Finally, we combined the results of both analyses and identified five ion channels (Kv7.2, Kv1.1, Nav1.7, Nav1.8, and TRPA1) and two molecular processes (G_α*q*_-coupled receptor phosphorylation and Nav1.7 and Nav1.8 phosphorylation) whose modifications could potentially regulate hyperalgesic priming of mouse muscle nociceptors.

### Analysis of modification of model-identified key proteins and molecular processes

**FIGURE 7:**
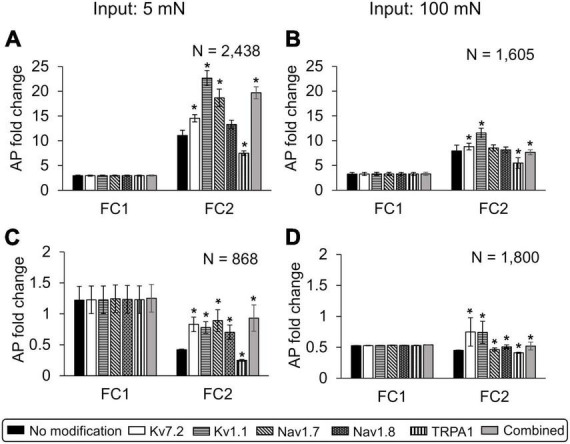
*In silico* analysis identified the relative contributions of specific neuroplastic changes in model-identified key ion channels to action potential (AP) generation in primed and non-primed neurons. We simulated an individual twofold expression increase in Nav1.7, Nav1.8, and TRPA1; an individual twofold expression decrease in Kv7. 2 and Kv1.1; as well as simultaneous modification of Kv7.2, Nav1.7, Nav1.8, and TRPA1 (gray bars) after the first exposure to an inflammatory event in primed and non-primed neuron sets identified during global sensitivity analysis. We compared the means and one standard error (SE) of the AP fold changes (FC1 and FC2) in response to 5 and 100 mN forces, after two subsequent inflammatory mediator exposures derived from simulations implementing the six modifications involving the individual and combined increase or decrease in expression of proteins with corresponding simulations with no modifications (i.e., the solid bars) in the **(A,B)** primed and **(C,D)** non-primed neuron groups. Solid bars in all panels indicate the means and one SE of the magnitude of AP fold change in simulations with no modification. Because the data from all the groups were not normal (which we established by performing a Kolmogorov–Smirnov test), we used a Wilcoxon rank sum test, which is typically used to compare the means of two independent samples when we cannot assume normality, to determine if any of the protein modification significantly changed AP firing. An asterisk (*) indicates that the mean AP fold change due to a particular modification was significantly different from the simulations with no modification, with *p* ≤ 0.01.

To quantify the extent to which different inflammation-induced neuroplastic modifications in membrane proteins affect subsequent neuronal response, we performed simulations where we modified each model-identified key protein, one at a time, after the first inflammatory event in the primed and non-primed neuron groups for both the 5 and 100 mN force inputs. Specifically, for the three proteins Nav1.7, Nav1.8, and TRPA1, we simulated the effect of their overexpression, and for Kv1.1 and Kv7.2, we simulated the effect of reducing their expression on AP firing. We used these specific modifications based on our model results and literature evidence (Nicol et al., 1997; Blair and Bean, 2002; Kwan et al., 2009; Hameed, 2019). We also performed simulations where we combined all protein modifications to occur simultaneously. We induced these modifications after the first inflammatory event, i.e., at the 48-h simulation time point. We then compared the mean AP fold changes (FC1 and FC2) due to each of these modifications to the corresponding value in the simulations with no modifications (i.e., the baseline sensitization) (Figure 7).

Of the simulations with protein modifications, we identified 2,438 and 868 that ran successfully in the primed and non-primed neuron groups, respectively, when 5 mN force was used as input. When 100 mN was used as the force input, we identified 1,605 and 1,800 simulations that ran successfully in the primed and non-primed neuron groups, respectively. When either an innocuous (i.e., 5 mN) or a noxious (i.e., 100 mN) force was used as input, in the set of primed neurons (i.e., neurons in which subsequent inflammatory events increased AP firing without any modification), modifying TRPA1 (Figures 7A, B, vertically dashed bar for FC2), Kv1.1 (Figures 7A, B, horizontally dashed bar for FC2), and Kv7.2 (Figures 7A, B, white bar for FC2) expression as well as the combined modification of all four key proteins (Figures 7A, B, gray bar for FC2) significantly increased the average AP fold change after the second inflammatory event compared to the simulations with no modifications (Figures 7A, B, black bar for FC2). Of note, while the simulations captured the extent to which distinct neuroplastic modifications induced by hyperalgesic priming affected the neuron’s response to subsequent inflammatory events, the effect was quantitatively higher (for all modifications) when an innocuous force was used as input compared to a noxious force (Figures 7A, B, FC2 values).

In the set of non-primed neurons (i.e., neurons in which subsequent inflammatory events decreased AP firing), when either an innocuous force (i.e., 5 mN) or a noxious force (i.e., 100 mN) was used as input, all the implemented neuroplastic modifications significantly increased the average AP fold change after the second inflammatory event (Figures 7C, D) compared to the simulations with no modifications (Figures 7C, D, black bar for FC2). With the exception of Kv1.1 and Kv7.2 expression reduction (Figure 7D, open and horizontally striped bars for FC2), as observed in the primed neuron group, the AP fold changes induced by the different protein modifications after exposure to the second inflammatory event were higher for the innocuous force input (Figure 7C) than for the noxious force (Figure 7D). Overall, based on our simulation results, hyperalgesic priming involving Nav1.7 and Kv1.1 neuroplasticity (compared to Nav1.8, Kv7.2, and TRPA1) appeared to have the largest effect on the sensitivity of muscle nociceptors to mechanical forces during multiple inflammatory events.

## Discussion

Hyperalgesic priming in peripheral nociceptors causes them to become hyperexcitable to subsequent injuries or inflammatory events when they have experienced a prior sensitization event, thus preserving a “memory” of the prior event (Woolf and Ma, 2007; Reichling and Levine, 2009). Hyperalgesic priming could potentially lead to the transition of acute to chronic pain, however, its underlying mechanisms are not well understood (Parada et al., 2005; Kandasamy and Price, 2015) although such knowledge might hold the key to developing therapies to prevent or reverse pain chronification. Inflammatory mediators present during injury sensitize nociceptors by decreasing their activation thresholds and increasing their responsiveness (i.e., AP firing) to stimuli. This increase reflects changes in the expression and function of the nociceptor’s membrane proteins. However, we still do not know which ion channels, pumps, and exchangers are key for the generation of AP responses after multiple inflammatory events. In this study, we used computational analysis to identify such key proteins and molecular processes. We first extended our validated, multi-compartment computational model of a mouse muscle nociceptor to incorporate Epac-mediated PKC activation (Parada et al., 2005) and the kinetics of a mechanosensitive ion channel, TRPV4. The extended model accounts for the activity of 18 membrane proteins, four ER membrane proteins, and 29 second-messenger molecules, including proteins, molecules, kinases, as well as Na^+^, K^+^, and Ca^2+^ ions, and describes 46 intracellular processes, including the activation and inactivation of the various membrane proteins as well as intracellular proteins and molecules by inflammatory mediators. We then calibrated and validated the model using experimental data capturing the effects of two subsequent inflammatory events on the AP responses in rat nociceptors. In agreement with experimental observations, our simulations of two subsequent inflammatory events predicted an increase in AP firing and a reduction in a nociceptor’s mechanical activation threshold with each inflammatory event (Figures 3B, C).

To identify key regulators of the AP response, we used the model to simulate pain signaling responses to mechanical forces and two inflammatory events in 50,000 unique muscle nociceptors intended to represent the heterogeneity in protein expression and activity and the numerous plausible protein interactions that can occur *in vivo*. We found that modifications to five ion channels (Kv7.2, Kv1.1, Nav1.7, Nav1.8, and TRPA1) and two molecular processes (G_α*q*_-coupled receptor phosphorylation and Nav1.8 and Nav1.7 phosphorylation) strongly regulated the increase in the total number of APs fired by the neurons in response to both an innocuous (5 mN) and noxious (100 mN) mechanical force during inflammation. Moreover, by separately simulating the specific modifications in the expression of each of the five proteins, we showed that modifying the expression of Nav1.7 or Kv1.1 after the first inflammatory event significantly regulated the magnitude of AP firing. Therefore, Kv1.1 and Nav1.7 channels (specifically modification of their expression or activation) could be considered as potential targets for regulating the increased AP response of nociceptors due to hyperalgesic priming that occurs during musculoskeletal trauma.

### Key membrane proteins and molecular processes that regulate AP generation in primed neurons

Because a majority of the pathways activated by distinct inflammatory mediators converge within the neuron to increase the intracellular concentrations of PKC and PKA, various membrane-bound and intracellular molecules and proteins involved in PKC and PKA signaling have been investigated for their roles in hyperalgesic priming (Ferrari et al., 2013b; Gangadharan and Kuner, 2013; Araldi et al., 2016a,b; Fang et al., 2021). In fact, numerous behavioral studies have demonstrated that activation of PKC*ε* can consistently induce hyperalgesic priming in rats after multiple exposures to inflammatory mediators. The studies demonstrated that the generation of hyperalgesic priming in rat nociceptors depended on proteins downstream of PKC activation, such as cytoplasmic polyadenylation element-binding protein (CPEB), a protein responsible for translation of mRNA in the peripheral terminals of nociceptors. Furthermore, inhibiting downstream targets of CPEB (e.g., α calmodulin-dependent protein kinase II) or inhibiting nuclear transcription factors (e.g., via the administration of an oligodeoxynucleotide antisense) attenuated priming in rats (Aley and Levine, 1999; Reichling and Levine, 2009; Ferrari et al., 2013a,b, 2015; Araldi et al., 2016a). While we do not explicitly model molecules downstream of PKC activation that are involved in the mRNA translation of different membrane proteins, our PRCC analysis identified molecules that are precursors to PKC activation, i.e., G_α*q*_-coupled receptors phosphorylation and PIP_2_ activation, as key for increased AP firing in sensitized muscle neurons during a second inflammatory event (Figures 5C, D, black bars), further demonstrating the importance of PKC signaling in inflammation-induced neuronal response changes. However, because a behavioral pain response (i.e., paw withdrawal) was used as an indicator of the change in the neuronal response induced by hyperalgesic priming in the above-mentioned experimental studies, we still do not have electrophysiological correlates to this observed reduction in mechanical input thresholds. To develop new therapies that target the peripheral neurons, we need to understand how their response (i.e., AP firing) as well as the expression and function of key membrane proteins change during multiple inflammatory events.

Many studies that do measure electrophysiological responses of nociceptors have focused on the ability of PKC and PKA to evoke a change in the magnitude of neuronal AP firing by modifying the gating properties of key Na^+^ and K^+^ ion channels (already present on the neuronal membrane), such as Nav1.8 and Nav1.7 (Vijayaragavan et al., 2004; Wu et al., 2012; Tanaka et al., 2016), Kv1.1 (Nicol et al., 1997; King et al., 2014; D’Adamo et al., 2020), and TRPA1 (Kwan et al., 2006; Karashima et al., 2009; del Camino et al., 2010), among others, via phosphorylation. Therefore, a number of studies have investigated Nav1.8 and Nav1.7 modulation in inflammatory pain. For example, nerve growth factor (NGF)-induced thermal hyperalgesia was inhibited in Nav1.8 knockout (KO) mice compared to saline-treated mice (Kerr et al., 2001), and Nav1.8 inhibition via a pharmaceutical blocker showed reduced excitability in inflammatory and neuropathic rat models of pain (Payne et al., 2015). In addition, nociceptor-specific Nav1.7 KO mice showed an increased thermal and mechanical pain threshold and a decrease in AP response to various inflammatory mediators, including complete Freund’s adjuvant, PGE_2_, and NGF (Nassar et al., 2004). However, all these studies reported changes in neuronal responses due to modification of ion channels after only one inflammatory event. Therefore, we do not know if and for how long the inflammation-induced changes in the expression and function of these channels last, because none of the studies measured the neuronal responses after a second inflammatory event.

Our analysis allowed us to characterize the neuronal AP responses after two inflammatory events in two distinct scenarios, one where we pre-exposed a group of neurons to an innocuous force (5 mN) and another where we pre-exposed them to a noxious force (100 mN), both after the administration of an inflammatory mediator. We found that, while the number of APs fired were, on average, higher for the 100 mN force input compared to 5 mN, the AP fold changes, especially after the second inflammatory event, were higher when the neurons were stimulated by an innocuous (i.e., 5 mN) force input, suggesting that the effect of hyperalgesic priming on the increase in the neurons’ excitability was higher when the initial force inputs were innocuous. Furthermore, our ability to simulate two inflammatory events led to the identification of proteins and processes key for initiating hyperalgesic priming (i.e., after the first inflammation event) and for regulating it. For example, in agreement with previous electrophysiological studies, our analysis identified Nav1.7, TRPA1, and Kv1.1 as key in driving the increase in the magnitude of AP firing after a single inflammatory event (Figures 5A, C). In addition to these three proteins, our analysis also identified Nav1.8 and Kv7.2 as well as molecular processes of phosphorylation of G_α*q*_-coupled receptor, Nav1.8, and Nav1.7 as key for the increase in AP firing after a second inflammatory event (Figures 5B, D), which current experimental studies did not measure. Overall, our computational analyses identified five proteins that were key for the increase in AP firing to mechanical forces after two subsequent inflammatory events.

### Relative contributions of model-identified key proteins/processes

While previous studies and our computational analysis have highlighted the role of individual ion channels or protein kinases in regulating pain responses during multiple inflammatory events (Nicol et al., 1997; Baker, 2005; Kwan et al., 2009; Gold and Gebhart, 2010; King et al., 2014; Nagaraja et al., 2021), we were able to compare the relative effect of modifying different proteins and kinases, one at a time, on AP firing in the same set of 50,000 simulated neurons, which is not feasible in experimental studies. For example, in a group of 2,438 distinct primed neurons, with no specific modification implemented, the average inflammation-induced increase in AP fold change in response to 5 mN force was 3.1 (SE = 0.18) and 11.1 (SE = 1.03) after the first and second inflammatory events, respectively. In the same neurons, five of the six distinct modifications we implemented (involving the proteins identified as key by our GSA), one at a time, significantly altered the average AP fold change after the second inflammatory event (Figure 7A). Of these five modifications, three increased the AP fold change by greater than 70%. Specifically, a twofold decrease in Kv1.1 expression, a twofold increase of Nav1.7 expression, and a combined modification of Kv7.2, Nav1.7, Nav1.8, and TRPA1 increased the average AP fold change by 104, 70, and 77%, with mean FC2 values of 22.6 (SE = 1.7), 18.5 (SE = 1.7), and 19.4 (SE = 1.2), respectively (Figure 7A, open, diagonally dashed, and gray bars). We saw similar results when we simulated the modifications in another group of 1,605 primed neurons using 100 mN force as input (Figure 7B). We can use this information while designing new strategies to regulate the effect of hyperalgesic priming. For example, since the modifications of both Kv1.1 and Nav1.7 individually had a significant effect on AP fold change compared to other protein modifications, we could test them as potential targets to regulate the degree of hyperalgesic priming and select the one with the least side effects.

We also simulated the neuroplastic modifications in the non-primed neuron group. We assumed that these simulations represented silent nociceptors, or non-responding sensory neurons, and aimed to determine whether there were any specific neuroplastic modifications initiated in these neurons by the first inflammatory event that could activate them to sensitize during a second inflammatory event. For example, a group of 1,800 neurons that initially (with no modifications) showed no increase in mean AP fold change after the first or second inflammatory events [Figure 7D, black bars, FC1 and FC2 of 0.50 (SE = 0.004) and 0.45 (SE = 0.004), respectively] became sensitized (or acquired nociceptive properties) after the second inflammatory event when we decreased Kv7.2 or Kv1.1 expression after the first inflammatory event. [Figure 7D, open and horizontally dashed bars, FC2 of 0.75 (SE = 0.20) and 0.74 (SE = 0.17), respectively]. Thus, in addition to identifying a panel of key proteins and molecules that could be potential targets for regulating the neuronal excitability after inflammation, our analysis also provided a one-to-one comparison of the efficacy of targeting each key protein in the same population of neurons.

### Assumptions and limitations

Our computational model has several limitations arising from simplifying assumptions required to capture the complex nature of hyperalgesic priming in muscle afferent neurons. First, due to limited availability of electrophysiological data for the effects of multiple inflammatory events on AP firing in mice, we used data from rat neurons to calibrate and validate the model, which might affect model accuracy. Second, because we only represent two specific GPCR signaling pathways activated by a subset of the inflammatory mediators that can be present in an injured tissue, it is possible that proteins and molecules involved in other pathways not currently accounted for might be important for regulating hyperalgesic priming. However, as previously discussed, many inflammatory pathways are known to converge within the neuron, resulting in an increase in the concentrations of PKC and PKA (Gold and Flake, 2005; Gold and Gebhart, 2010; Gangadharan and Kuner, 2013; Pak et al., 2018), whose effects, including Epac-mediated PKC activation, we do currently model. Therefore, we could incorporate the effects of other inflammatory mediators if and when relevant. Third, in our model, we adopted many parameter values from previous computational studies developed to describe neurons from animals other than mice or from physiological tissues other than muscle (Bennett et al., 2005; Lindskog et al., 2006; Mandge and Manchanda, 2018). While we performed a validation procedure to match our computational simulations to experimental data recorded from rat neurons, we did not directly derive parameters from single ion channel current measurements in mouse neurons. This simplification could impact the accuracy of certain model parameters. Fourth, we chose a 48-h period between the two subsequent inflammatory events based on experimental evidence that shows that the reduction in the mechanical threshold due to the addition of an inflammatory mediator typically recovers in ∼24 h (Hendrich et al., 2013). However, behavioral studies have demonstrated that the effect of priming can persist for up to 1 week (Ferrari et al., 2013b,^2015^; Araldi et al., 2016a,b). Therefore, the mechanisms underlying the priming effect might differ based on the timing of subsequent inflammatory events in the primed neurons, which we do not currently consider but could potentially account for in future analyses. Finally, our hypotheses regarding the contributions of Kv7.2, Kv1.1, Nav1.7, Nav1.8, and TRPA1 and both Nav1.8 and Nav1.7 phosphorylation to the hyperexcitability of muscle nociceptors after two inflammatory events stem solely from simulations. These hypotheses need to be tested using experiments involving electrophysiological (patch clamp or dynamic) recordings in mice, and potentially even using human induced pluripotent stem cells, where we separately modify each protein or process and assess the effect of the modification on AP firing in response to multiple, sequential inflammatory events. Ultimately, there is always the question of translatability of the hyperalgesic mechanisms across species. Since we cannot perform *in vivo* investigations on human nociceptors, mouse models provide the opportunity to use genetic approaches to investigate the molecular mechanisms of nociceptive signaling. In addition, both rodents and humans are known to have the ability to sensitize following repetitive injuries and similar functional organization of the spinal cord, making them a useful substitute for human nociceptors (Fitzgerald, 2005; Toossi et al., 2021).

## Conclusion

Identification of hyperalgesic priming mechanisms in muscle nociceptors is challenging due to the heterogeneity in muscle neuron subpopulations and the plethora of inflammatory mediators that can act upon them. In this study, we specifically focused on the effect of two subsequent PGE_2_-induced inflammatory events on mechanical nociception in muscle tissue. The results of our computational analyses allowed us to hypothesize that (1) Kv7.2, Kv1.1, Nav1.7, Nav1.8, and TRPA1 as well as phosphorylation of G_α*q*_-coupled receptors, Nav1.7, and Nav1.8 regulate the increase in AP firing magnitude in primed muscle nociceptors; (2) decreasing Kv1.1 expression causes the greatest increase in AP firing in primed neurons compared to individual modifications of Kv7.2, Nav1.7, Nav1.8, or TRPA1; and (3) an inflammation-induced decrease in Kv7.2 or Kv1.1 expression in silent nociceptors or non-responsive sensory neurons might cause them to become responsive and sensitize during a subsequent inflammatory event when stimulated by noxious mechanical forces.

We can use our findings to further the area of pain research in different ways. First, performing *in vivo* studies to experimentally test our computationally derived hypotheses could confirm the roles of specific proteins in hyperalgesic priming of nociceptors and lead to an improved understanding of chronic pain initiation in muscle tissue. In addition, we could use our findings to assess whether the same proteins that prime muscle neurons to inflammatory pain are also involved in different pathological pain scenarios, such as pain arising from other sensory processing disorders, e.g., fibromyalgia. Finally, by extending the computational model to include the kinetics of pharmaceutical drugs that act as inhibitors or enhancers for specific ion channels or receptors, we can predict their efficacy in regulating the effects of hyperalgesic priming on subsequent pain-inducing events in a dose-dependent manner.

## Data availability statement

The original contributions presented in this study are included in this article/Supplementary material, further inquiries can be directed to the corresponding author.

## Author contributions

SN: Conceptualization, Formal analysis, Investigation, Methodology, Software, Visualization, Writing – original draft, Writing – review and editing. ST: Conceptualization, Writing – review and editing. JR: Conceptualization, Supervision, Visualization, Writing – review and editing.
