# Supplementary material for: Predictive analytics identifies key factors driving hyperalgesic priming of muscle sensory neurons
Source: Front Neurosci. 2023 Oct 24;17:1254154. doi: 10.3389/fnins.2023.1254154 (PMC10629345; doi:10.3389/fnins.2023.1254154)
Supplement: Supplementary file 1 [file Data_Sheet_1.PDF]

SUPPLEMENTARY MATERIAL

Predictive analytics identifies key factors driving hyperalgesic priming of muscle sensory neurons

by Sridevi Nagaraja, Shivendra G. Tewari, and Jaques Reifman

Table S1. Model variable name, description, and initial value

Variable name	Description	Initial value	Variable name	Description	Initial value
$Nav1.8_m$	Activation constant of voltage-gated Nav1.8 channel	0	$TREK_m$	Activation constant of two-pore K^+ channel	0
$Nav1.8_h$	Inactivation constant of voltage-gated Nav1.8 channel	1	Ka_n	Activation constant of M-type K^+ channel	0
$Nav1.7_m$	Activation constant of voltage-gated Nav1.7 channel	0	Ka_{hfast}	Fast inactivation constant of M-type K^+ channel	1
$Nav1.7_h$	Inactivation constant of voltage-gated Nav1.7 channel	1	Ka_{hslow}	Slow inactivation constant of M-type K^+ channel	1
$Nav1.9_m$	Activation constant of voltage-gated Nav1.9 channel	0	$Kv7_n$	Activation constant of voltage-gated Kv7.2 channel	0
$Nav1.9_h$	Inactivation constant of voltage-gated Nav1.9 channel	1	KDR_n	Activation constant of delayed rectifying K^+ channel	0
$Piezo_m$	Fast activation constant of Piezo2 channel	0	$BKCa_n$	Activation constant of large-conductance Ca^{2+}-activated K^+ channel	0
$Piezo_h$	Inactivation variable of Piezo2 channel	1	CaL_m	Activation constant of L-type voltage-gated Ca^{2+} channel	0
$ASIC3_m$	Activation constant of ASIC3 channel	0	CaL_h	Inactivation constant of L-type voltage-gated Ca^{2+} channel	1
$ASIC3_h$	Inactivation constant of ASIC3 channel	1	CaT_m	Activation constant of T-type voltage-gated Ca^{2+} channel	0
$TRPA1_m$	Activation constant of TRPA1 channel	0	CaT_h	Inactivation constant of T-type voltage-gated Ca^{2+} channel	1
$TRPA1_h$	Inactivation constant of TRPA1 channel	1	V_m	Membrane potential	-55 mV
$[Na_i^+]$	Intracellular Na^+ concentration	14 mM	$[K_i^+]$	Intracellular K^+ concentration	140 mM
$[Ca_i^{2+}]$	Intracellular free calcium ion concentration	5×10^{-5} mM	$[Ca_{ER}^{2+}]$	Calcium concentration in the endoplasmic reticulum	0.25 mM
IP_3	Intracellular inositol trisphosphate concentration	1×10^{-5} mM	hIP_3	Activation constant of the IP_3 receptor	0.667

$G_{\alpha qunphos}$	Total number of unphosphorylated $G_{\alpha q}$ receptors	1.7×10^{5}	$[G_{\alpha sact}]$	Concentration of α subunits of $G_{\alpha s}$ receptors	0
$G_{\alpha qphos}$	Total phosphorylated $G_{\alpha q}$ receptors	0	$[G_{\alpha s\beta\gamma}]$	Concentration of αβ subunits of $G_{\alpha s}$ receptors	0
$G_{\alpha q}$	Number of α subunits of $G_{\alpha q}$ receptors	0	$[G_{\beta\gamma s}]$	Concentration of β subunits of $G_{\alpha s}$ receptors	0
[DAG]	Intracellular diacylglycerol concentration	0 mM	[cAMP]	Intracellular concentrations of cyclic adenosine monophosphate	0 mM
PIP_2	Total number of phosphatidylinositol 4,5-bisphosphate	5×10^{7}	[RC]	Intracellular concentration of inactive PKA	2.2×10^{-4} mM
PLC	Concentration of active phospholipase C	0 mM	$[R_{cAMP}]$	Intracellular concentration of PKA regulatory subunit	0 mM
$[DAG_{PKC}]$	Intracellular concentration of DAG-PKC complex	0 mM	$[RC_{caMP2}]$	Intracellular concentration of intermediate regulatory PKA catalytic subunit	0 mM
PLC_{inact}	Concentration of membrane-bound inactive phospholipase C	1×10^{-2} mM	$[RC_{cAMP4}]$	Intracellular concentration of intermediate regulatory PKA-PKA catalytic subunit	0 mM
$[PKC_{inact}]$	Intracellular concentration of inactive protein kinase C	1×10^{-3} mM	[PKA]	Intracellular concentration of active protein kinase A	0 mM
[PKC]	Intracellular concentration of protein kinase C	0 mM	$V_{actNav1.7}$	Membrane potential threshold for Nav1.7 activation	-25.8 mV
$V_{actNav1.8}$	Membrane potential threshold for Nav1.8 activation	-11.4 mV	$V_{inactNav1.7}$	Membrane potential threshold for Nav1.7 inactivation	55.8 mV
$V_{inactNav1.8}$	Membrane potential threshold for Nav1.8 inactivation	-24.6 mV	$M_{actTRPA1}$	Mechanical force threshold for TRPA1 activation	40 mN
$V_{actKv1.1}$	Membrane potential threshold for Kv1.1 activation	-35 mV	$TRPV4_m$	Activation constant of TRPV4 channel	0
$TRPV4_h$	Inactivation constant of TRPV4 channel	1	$Epac_{inact}$	Intracellular concentration of inactive Epac	5×10^{-3} mM
$Epac_{act}$	Intracellular concentration of active Epac	0			

Table S2. Model parameter number (used in the model), name, description, value, units, and sources

P#	Parameter name	Description	Value	Unit	References
**Na^+-Ca^{2+}exchanger**					
1	kNCX	Constant for NCX	25.85		(Nagaraja et al., 2021)
2	kNa	Half-saturation constant for extracellular [Na^+]	87.50	mM	
3	kCa	Half-saturation constant for extracellular [Ca^{2+}]	1.38	mM	
4	$Imax_{NCX}$	Maximum current density	1×10^5	nS	
**ASIC3 channel**					
5	Vh_{ASIC}	Half-activation pH for activation factor	6.202	pH	(Nagaraja et al., 2021)
6	$k_{act}ASIC$	Steepness factor of activation	0.1754	pH	
7	τact_{ASIC}	Hill slope of activation factor	5.000	ms	
8	Vs_{ASIC}	Half-inactivation pH for activation factor	7.061	pH	
9	$k_{inact}ASIC$	Steepness factor of inactivation	0.0452	pH	
10	$Imax_{ASIC1\beta}$	Maximum conductance of ASIC channel	15.0	nS	
**Na^+-K^+ pump**					
11	nH_{Na}	Hill coefficient for sodium and potassium	1.5		(Nagaraja et al., 2021)
12	KNa_{NaK}	Binding constant for intracellular [Na^+]	14.5	mM	
13	KK_{NaK}	Binding constant for extracellular [K^+]	1.5	mM	
14	$Imax_{NaK}$	Maximum current density	150	pA	
**Piezo2 channel**					
15	Vh_{Piezo}	Half-activation force for inactivating factor	0.9	mN	(Nagaraja et al., 2021)
16	Vs_{Piezo}	Half-activation force for inactivating factor	0.6	mN	
17	$k1_{inact}Piezo$	Steepness factor of inactivation	0.3	mN	
18	$k2_{inact}Piezo$	Steepness factor of inactivation	0.1	mN	
19	τact_{Piezo}	Time constant for activation factor	1	ms	
20	$\tau 1inact_{Piezo}$	Time constant for fast inactivation factor	3	ms	
21	$Imax_{Piezo}$	Maximum conductance of Piezo channel	40	nS	
**TREK-1 channel**					
22	Vm_{TREK}	Half-activation force for activation factor	8	mN	(Nagaraja et al., 2021)
23	$kact_{TREK}$	Steepness factor of activation	1	mN	
24	τact_{TREK}	Activation time constant	1	ms	
25	$Imax_{TREK}$	Maximum TREK channel conductance	0.5	nS	
**TRPA1 channel**					
26	$kact_{TRPA1}$	Steepness factor of activation	20	mN	(Nagaraja et al., 2021)
27	Vh_{TRPA1}	Half-activation force for inactivation factor	40	mN	
28	$kinact_{TRPA1}$	Steepness factor of inactivation	20	mN	
29	τact_{TRPA1}	Activation time constant	1	ms	
30	$\tau inact_{TRPA1}$	Inactivation time constant	5	ms	
31	$Imax_{TRPA1}$	Maximum conductance	15	nS	
**Kv7.2**					
32	$Imax_{Kv7}$	Maximum Kv7 conductance	600	nS	(Nagaraja et al., 2023)
33	$k1act_{Kv7}$	Steepness factor of activation	0.00395	mV	(Nagaraja et al., 2021)
34	Vm_{Kv7}	Half-activation membrane potential for activation factor	15	mV	
35	$k2act_{Kv7}$	Steepness factor of activation	40	mV	
36	$k1inact_{Kv7}$	Steepness factor of inactivation	0.00395	mV	
37	$k2inact_{Kv7}$	Steepness factor of inactivation	20	mV	

38	τact_{Kv7}	Activation time constant	3	ms	(Nagaraja et al., 2023)
**Kv1.1 channel**					
39	$Imax_{KDR}$	Maximum KDR conductance	200	nS	(Nagaraja et al., 2021)
40	δ_{KDR}	Activation factor	0.577	mV	
41	$kact_{KDR}$	Steepness factor of activation	15.4	mV	
42	τact_{TRPA1}	Activation time constant	300	ms	(Nagaraja et al., 2023)
**A-type K^+ channel**					
43	$Imax_{Ka}$	Maximum A-type K^+ channel conductance	6	nS	(Nagaraja et al., 2023)
**Nav1.8 channel**					
44	$Imax_{Nav1.8}$	Maximum Nav1.8 channel conductance	150	nS	(Nagaraja et al., 2023)
45	$kact_{Nav1.8}$	Steepness factor of activation	8.75	mV	(Nagaraja et al., 2021)
46	$kinact_{Nav1.8}$	Steepness factor of inactivation	5.76	mV	
**Nav1.9 channel**					
47	$Imax_{Nav1.9}$	Maximum Nav1.9 channel conductance	0.5	nS	(Nagaraja et al., 2023)
**Nav1.7 channel**					
48	$Imax_{Nav1.7}$	Maximum Nav1.7 channel conductance	212.0	nS	(Nagaraja et al., 2021)
49	$kact_{Nav1.7}$	Steepness factor of activation	7.8	mV	
50	$kinact_{Nav1.7}$	Steepness factor of inactivation	8.9	mV	
**Potassium leak channel**					
51	$Imax_{Kleak}$	Leak channel conductance	0.6	nS	(Nagaraja et al., 2023)
**Ca^{2+}-activated K^+ channel**					
52	$Imax_{BKCa}$	Maximum BKCa channel conductance	10	nS	(Nagaraja et al., 2021)
**L-type voltage-gated Ca^{2+} channel (VGCC)**					
53	$Imax_{LCa}$	Maximum L-type VGCC conductance	10	nS	(Nagaraja et al., 2021)
54	Vm_{LCa}	Half-activation potential for activation factor	-22.8	mV	
55	$kact_{LCa}$	Steepness factor for activation	9.85	mV	
56	Vh_{LCa}	Half-activation potential for inactivation factor	-34.61	mV	
57	$kinact_{LCa}$	Steepness factor for inactivation	5.95	mV	
58	τact_{LCa}	Time constant for activation	2.38	ms	
59	$\tau inact_{LCa}$	Time constant for inactivation	25.2	ms	
**T-type voltage-gated Ca^{2+} channel**					
60	Vm_{TCa}	Half-activation potential for activation factor	-25.0	mV	(Nagaraja et al., 2021)
61	$Kact_{TCa}$	Steepness factor for activation	-5.0	mV	
62	Vh_{TCa}	Half-activation potential for inactivation factor	-38.0	mV	
63	$Kinact_{TCa}$	Steepness factor for inactivation	-5.0	mV	
64	τact_{TCa}	Time constant for activation	1	ms	
65	$\tau inact_{TCa}$	Time constant for inactivation	409	ms	
66	$Imax_{TCa}$	Maximum T-type VGCC conductance	0.099	nS	
**IP_3 receptor (IP_3R)**					
67	$Imax_{IP3R}$	Rate constant of Ca^{2+} release by IP_3R	0.00288	nS	(Nagaraja et al., 2021)
68	$kdis_{IP3}$	Dissociation constant for IP_3 binding to IP_3R	2.7	mM	
69	$kdisinact_{Ca}$	Dissociation constant for Ca^{2+} inactivation	1.0×10^{-4}	mM	

70	$kdisact_{Ca}$	Dissociation constant for Ca^{2+} activation	1.7×10^{-4}	mM	
71	k_{Ca}	Rate of Ca^{2+} binding to the inhibitory site	0.0003	mM s^{-1}	
**PMCA pump**					
72	$Imax_{PMCA}$	Maximum current	7.6418	pA	(Nagaraja et al., 2021)
73	KCa_{PMCA}	Michaelis constant	0.1562	mM	
**SERCA pump**					
74	KCa_{SERCA}	Michaelis constant	3.94×10^{-4}	mN	(Nagaraja et al., 2021)
75	$Imax_{SERCA}$	Maximum SERCA uptake	0.002	pA	
**Ryanodine receptor (RyR)**					
76	KCa_{CICR}	Minimum intracellular [Ca^{2+}] for RyR activation	1.2×10^{-4}	mM	(Nagaraja et al., 2021)
77	$Imax_{CICR}$	Rate constant of Ca^{2+} release by RyR	5.03×10^{-4}	pA	
78	kd_{CICRCa}	Dissociation constant for Ca^{2+} inactivation	0.0501	mM	
**Endoplasmic reticulum (ER) Ca^{2+} leak**					
79	$Imax_{ERleak}$	Maximum passive leak from ER	3.03×10^{-5}	pA	(Nagaraja et al., 2021)
**Calcium buffering in ER**					
80	K_{CQSN}	Binding affinity of calsequestrin	1.21	mM	(Nagaraja et al., 2021)
81	CQSN	Concentration of calsequestrin in ER	16.0	mM	
**Protein kinase C (PKC) activation and signaling**					
82	RTG	Total unphosphorylated $G_{\alpha q}$ receptors	2.00×10^{4}		(Bennett et al., 2005; Mohan et al., 2017)
83	K_{1G}	Unphosphorylated receptor dissociation constant	0.01	mM	
84	K_{2G}	Phosphorylated receptor dissociation constant	0.2	mM	
85	k_{rG}	Receptor recycling rate	1.75×10^{-7}	ms^{-1}	
86	k_{pG}	Receptor phosphorylation rate	1.00×10^{-3}	ms^{-1}	
87	k_{eG}	Receptor endocytosis rate	6.00×10^{-6}	ms^{-1}	
88	$epsilon_{G}$	Fraction of mobile receptors	0.85		
89	GTG	Total activated $G_{\alpha q}$ receptors	1.00×10^{5}		
90	k_{degG}	IP_3 degradation rate	0.00125	ms^{-1}	
91	k_{aG}	$G_{\alpha q}$ subunit activation rate	1.70×10^{-4}	ms^{-1}	
92	k_{dG}	$G_{\alpha q}$ subunit deactivation rate	1.50×10^{-3}	ms^{-1}	
93	$PIP2_{T}$	Total PIP_2 molecules	5.00×10^{7}		
94	r_{rG}	PIP_2 replenishment rate	1.50×10^{-5}	ms^{-1}	
95	k_{cG}	Dissociation constant for Ca^{2+} binding to PLC	4.00×10^{-4}	mM	
96	α_{G}	Effective signal gain parameter	2.78×10^{-8}	ms^{-1}	
97	γ_{G}	Coefficient to convert number of molecules to concentration	6.00×10^{8}		
98	k_{PLCact}	PLC activation rate	2.2967	$mM^{-1}\cdot ms^{-1}$	(Nagaraja et al., 2023)
99	$k_{PLCinact}$	PLC inactivation rate	0.3389	ms^{-1}	
100	k_{hyd}	Rate of diacylglycerol (DAG) activation by PIP_2	4.99×10^{-10}	$mM^{-2}\cdot ms^{-1}$	(Mohan et al., 2017)
101	k_{deg}	DAG degradation rate	0.0499	ms^{-1}	
102	k_{actPKC}	Rate of PKC activation by DAG	0.20	$mM^{-1}\cdot ms^{-1}$	(Nagaraja et al., 2023)
103	$k_{inactPKC}$	PKC inactivation rate	0.0022	ms^{-1}	
104	k_{off}	Rate of dissociation of DAG-PKC complex	8.00×10^{-3}	ms^{-1}	
105	k_{dp}	Rate of association of DAG and PKC	0.0112	ms^{-1}	
106	$k_{EpacPKC}$	Rate of PKC activation by Epac	4.50×10^{-9}	$mM^{-2}\cdot ms^{-1}$	Modified
**Protein kinase A (PKA) activation and signaling**					
107	AC_{tot}	Total basal concentration of adenyl cyclase	2.90×10^{-5}	mM	(Leander and Friedman, 2014)
108	$GPCR_{tot}$	Basal concentration of total phosphorylated $G_{\alpha s}$ receptors	9.70×10^{-6}	mM	

109	$G_{\alpha stot}$	Total activated of $G_{\alpha s}$ subunits	0.0061		
110	k_{EPdiss}	Dissociation rate constant	1.90×10^{-5}		
111	$k1_{G\alpha s}$	$G_{\alpha s}$ activation rate	5.00×10^{-3}	ms^{-1}	
112	$k2_{G\alpha s}$	$G_{\alpha s}$ hydrolysis rate	7.00×10^{-5}	ms^{-1}	
113	$k3_{G\alpha s}$	$G_{\alpha s}$-βγ association rate	0.7	$mM^{-1}\cdot ms^{-1}$	
114	$k4_{G\alpha s}$	$G_{\alpha s}$-βγ disassociation rate	1.89×10^{-5}	ms^{-1}	
115	k_{ACdiss}	Dissociation rate constant	3.60×10^{-6}	mM	
116	$kAC_{\beta\gamma diss}$	Disassociation constant for AC and βγ	9.00×10^{-5}	mM	
117	$k5_{G\alpha s}$	Active cAMP production rate	0.0105	ms^{-1}	
118	$k6_{G\alpha s}$	Basal cAMP production rate	3.5×10^{-4}	ms^{-1}	
119	$k_{degcAMP}$	cAMP degradation rate	0.013	ms^{-1}	
120	K_{fPKA}	Rate of association of PKA and cAMP	1.30×10^{-2}	$mM^{-2}\cdot ms^{-1}$	(Lindskog et al., 2006)
121	K_{bPKA}	Rate of disassociation of RCcaMP2	6.00×10^{-6}	ms^{-1}	
122	kf_9	Rate of association of cAMP and RCcaMP2	1.73×10^{-2}	$mM^{-2}\cdot ms^{-1}$	
123	kb_9	Rate of disassociation of RCcaMP4	6.00×10^{-5}	ms^{-1}	
124	kf_{10}	Rate of disassociation of RC and RCcaMP	8.00×10^{-6}	ms^{-1}	
125	kb_{10}	Rate of association of RC and RCcaMP	4.8	$mM^{-2}\cdot ms^{-1}$	
**TRPA1 phosphorylation by PKA and PKC**					
126	$k_{halfPKC}$	Phosphorylation factor of PKC	5.47×10^{-6}	mV	(Nagaraja et al., 2023)
127	$k_{slopePKC}$	Steepness factor of PKC	1.84×10^{-5}	mV	
128	$k_{halfPKA}$	Phosphorylation factor of PKA	3.47×10^{-5}	mV	
129	$k_{slopePKA}$	Steepness factor of PKA	8.39×10^{-6}	mV	
130	$\tau_{TRPA1phos}$	TRPA1 phosphorylation time constant	5×10^{6}	ms^{-1}	
131	$\tau_{Kv1.1phos}$	Kv1.1 phosphorylation time constant	5×10^{6}	ms^{-1}	
132	$\tau_{Navphos}$	Nav1.8 and Nav1.7 phosphorylation time constant	5×10^{6}	ms^{-1}	
**Epac kinetics**					
133	kf_{Epac}	Rate of Epac activation by cAMP	4.00×10^{-2}	$mM^{-1}\cdot ms^{-1}$	Modified
134	kb_{Epac}	Rate of Epac inactivation	1.00×10^{-7}	ms^{-1}	
**TRPV4 channel**					
135	Vm_{TRPV4}	Half-activation force for activation factor	120	mN	Modified
136	$kact_{TRPV4}$	Steepness factor of activation	40	mN	
137	Vh_{TRPV4}	Half-activation force for inactivation factor	110	mN	
138	$kinact_{TRPV4}$	Steepness factor of inactivation	40	mN	
139	τact_{TRPV4}	Activation time constant	1	ms	
140	$\tau inact_{TRPV4}$	Inactivation time constant	5	ms	
141	$Imax_{TRPV4}$	Maximum conductance	25	nS	

Figure S1

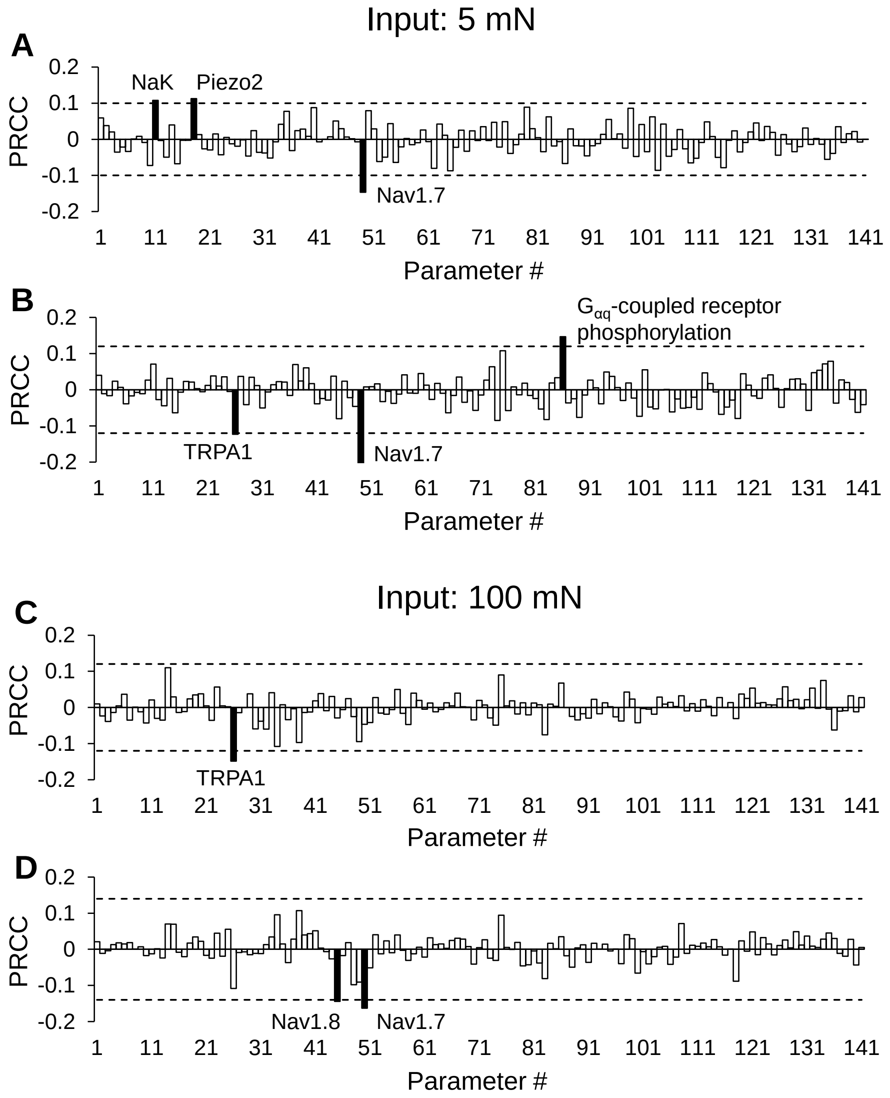

Figure S1. Partial rank correlation coefficient (PRCC) analysis identified key proteins and processes for action potential (AP) regulation. The bars show the PRCC values between the 141 model parameters and the fold changes in the number of APs fired after separate applications of the first (FC1) and second (FC2) inflammatory events in response to a mechanical force of (A and B) 5 mN and (C and D) 100 mN computed from non-primed neuron simulations. The PRCCs above their respective thresholds (dotted horizontal lines) that were statistically significant (i.e., $p < 0.01$) are indicated by solid black bars, and the labels of the bars show the ion channels/ion pumps or the rates of intracellular processes that these parameters describe in the model. The number of simulations in which the number of APs fired decreased after inflammation were 868 and 1,800 for applied mechanical forces of 5 and 100 mN, respectively.

**Table S3. List of Bhattacharyya coefficients for the model's 141 parameters, computed using the parameter distributions in the simulations of primed and non-primed neurons with 5 mN as the mechanical force input.**					
**Parameter number**	**Bhattacharyya coefficient**	**Parameter number**	**Bhattacharyya coefficient**	**Parameter number**	**Bhattacharyya coefficient**
1	98.316	48	99.0022	95	99.06164
2	98.74842	49	90.66936	96	99.12164
3	98.79099	50	98.94895	97	99.41468
4	99.23742	51	98.71262	98	98.7011
5	99.18448	52	98.92985	99	98.86731
6	98.99486	53	98.98166	100	98.78743
7	99.17083	54	99.0296	101	99.00348
8	99.12464	55	98.79053	102	98.88179
9	99.08793	56	98.60969	103	98.94124
10	98.86125	57	98.77831	104	98.8523
11	98.30111	58	99.40019	105	99.18612
12	99.03442	59	99.22227	106	98.83501
13	98.5347	60	98.61028	107	99.13678
14	98.68843	61	99.06888	108	98.97876
15	98.82275	62	98.78876	109	99.05859
16	99.11099	63	99.24041	110	98.96596
17	99.06757	64	99.01714	111	99.00675
18	98.61939	65	98.52615	112	98.96643
19	99.1394	66	99.29926	113	99.22244
20	98.98423	67	99.19684	114	99.15709
21	99.26381	68	99.05577	115	99.41608
22	98.80898	69	98.99935	116	98.94587
23	98.50743	70	98.77868	117	98.8429
24	99.15764	71	98.9439	118	98.87955
25	99.21153	72	99.46032	119	99.16869
26	97.80683	73	98.67016	120	98.90073
27	99.09572	74	98.82317	121	98.73387
28	99.05073	75	99.08261	122	99.11592
29	98.73503	76	99.17533	123	99.05045
30	98.96909	77	98.96563	124	99.07785
31	98.78362	78	99.18694	125	98.90792
32	98.90133	79	98.88288	126	99.05313
33	99.25065	80	99.27854	127	98.58357
34	99.16391	81	98.63718	128	98.94676
35	99.12019	82	98.55807	129	98.87249
36	98.80205	83	99.17205	130	99.0314
37	99.1157	84	99.1117	131	98.92054
38	98.91737	85	99.24393	132	98.74874
39	99.1677	86	98.89103	133	99.1226
40	97.61795	87	98.78263	134	97.98036
41	98.71703	88	98.63302	135	99.05137
42	99.08169	89	98.85289	136	98.88786
43	98.84852	90	99.25689	137	98.51743
44	98.6371	91	98.84508	138	99.07299
45	98.33291	92	99.18007	139	98.74111
46	98.54883	93	98.73777	140	99.14967
47	99.20523	94	99.2376	141	98.89896

**Table S4. List of Bhattacharyya coefficients for the model's 141 parameters, computed using the parameter distributions in the simulations of primed and non-primed neurons with 100 mN as the mechanical force input.**					
**Parameter number**	**Bhattacharyya coefficient**	**Parameter number**	**Bhattacharyya coefficient**	**Parameter number**	**Bhattacharyya coefficient**
1	98.97471	48	99.21922	95	99.28027
2	99.14769	49	95.45383	96	99.57322
3	99.08383	50	98.00699	97	99.00213
4	98.96578	51	99.50992	98	99.12387
5	99.28141	52	99.40834	99	99.33095
6	99.29721	53	99.34777	100	99.10484
7	99.37789	54	99.34557	101	99.14631
8	99.21188	55	99.17382	102	99.06913
9	99.00339	56	99.42108	103	99.3234
10	99.03597	57	99.16673	104	99.10137
11	98.86663	58	99.21102	105	99.35095
12	98.85612	59	99.42119	106	99.09851
13	98.85329	60	99.14152	107	99.15001
14	98.70583	61	99.11676	108	99.1934
15	99.139	62	99.35459	109	99.11457
16	98.97508	63	99.28529	110	99.39478
17	99.44321	64	99.3073	111	99.03244
18	99.35413	65	99.13625	112	99.15034
19	98.92529	66	99.47764	113	99.26723
20	99.22481	67	99.42363	114	99.0194
21	99.50815	68	99.45286	115	98.91033
22	99.42544	69	99.2623	116	99.03258
23	99.42974	70	99.15706	117	99.04007
24	99.19148	71	98.9901	118	99.47244
25	99.18853	72	99.17323	119	99.19174
26	94.53172	73	99.53708	120	99.32426
27	99.11393	74	98.96346	121	99.22546
28	99.44433	75	99.12346	122	99.3276
29	99.35687	76	99.41336	123	99.21091
30	99.48757	77	99.3406	124	99.25838
31	98.74267	78	99.09879	125	99.44502
32	98.89949	79	99.18878	126	99.28557
33	99.30681	80	99.43335	127	98.82338
34	98.86577	81	99.49493	128	99.38316
35	99.34283	82	99.08684	129	99.30928
36	99.33242	83	99.11029	130	99.02666
37	99.25947	84	99.17069	131	99.5521
38	98.303	85	99.08659	132	97.89117
39	99.17682	86	99.29433	133	99.06541
40	99.21178	87	99.52379	134	99.04732
41	99.25964	88	99.31336	135	97.17284
42	99.43335	89	99.25353	136	98.77796
43	99.19877	90	99.46747	137	98.94102
44	98.86352	91	99.167	138	99.18508
45	98.9485	92	99.29838	139	98.94011
46	99.36464	93	99.23199	140	99.0147
47	99.33456	94	99.30463	141	99.30628

Model equations for description of transmembrane currents, endoplasmic reticulum (ER) mechanisms, Nernst potentials, and mass balance of intracellular Na^+, K^+, and Ca^{2+} ions

Transmembrane mechanisms

1. Voltage-gated Nav1.8 channel

$$\frac{\mathrm{dNav1.8_m}}{\mathrm{d}t}=\frac{\mathrm{-Nav1.8_m+Nav1.8_{mss}}}{\tau_{\mathrm{m_{Nav1.8}}}}$$

$$\frac{\mathrm{dNav1.8_h}}{\mathrm{d}t}=\frac{\mathrm{-Nav1.8_h+Nav1.8_{hss}}}{\tau_{\mathrm{h_{Nav1.8}}}}$$

$$\mathrm{am_r}=\frac{7.2}{1+e^{((V_m-0.063)/7.86)}}$$

$$\mathrm{bm_r}=\frac{7.4}{1+e^{((V_m+53.06)/19.34)}}$$

$$\mathrm{ah_r}=0.003+\frac{1.63}{1+e^{((V_m+68.5)/10.01)}}$$

$$\mathrm{bh_r}=0.81-\frac{0.81}{1+e^{((V_m-11.44)/13.12)}}$$

$$\tau_{\mathrm{m_{Nav1.8}}}=\frac{1}{\mathrm{am_r+bm_r}}$$

$$\tau_{\mathrm{h_{Nav1.8}}}=\frac{1}{\mathrm{ah_r+bh_r}}$$

$$\mathrm{Nav1.8_{mss}}=\frac{1}{1+e^{\left(\frac{\mathrm{Vm_{Nav1.8}}-V_m}{\mathrm{kact_{Nav1.8}}}\right)}}$$

$$\mathrm{Nav1.8_{hss}}=\frac{1}{1+e^{\left(\frac{V_m+\mathrm{Vh_{Nav1.8}}}{\mathrm{kinact_{Nav1.8}}}\right)}}$$

$$I_{\mathrm{Nav1.8}}=\mathrm{Imax_{Nav1.8}}\cdot\mathrm{Nav1.8_m^2}\cdot\mathrm{Nav1.8_h}\cdot(V_m-V_{Na})$$

2. Voltage-gated Nav1.9 channel

$$\frac{\mathrm{dNav1.9_m}}{\mathrm{d}t}=\frac{\mathrm{-Nav1.9_m+Nav1.9_{mss}}}{\tau_{\mathrm{m_{Nav1.9}}}}$$

$$\frac{dNav1.9_h}{dt}=\frac{-Nav1.9_h+Nav1.9_{hss}}{\tau_{h_{Nav1.9}}}$$

$$am_{1.9}=\frac{1.548}{1+e^{((V_m-11.01)/-14.871)}}$$

$$bm_{1.9}=\frac{8.685}{1+e^{((V_m+112.4)/22.9)}}$$

$$ah_{1.9}=\frac{0.2574}{1+e^{((V_m+63.264)/3.719)}}$$

$$bh_{1.9}=\frac{0.54}{1+e^{((V_m+0.28)/-0.093)}}$$

$$\tau_{m_{Nav1.9}}=\frac{1}{am_{1.9}+bm_{1.9}}$$

$$\tau_{h_{Nav1.9}}=\frac{1}{ah_{1.9}+bh_{1.9}}$$

$$Nav1.9_{mss}=\frac{am_{1.9}}{am_{1.9}+bm_{1.9}}$$

$$Nav1.9_{hss}=\frac{ah_{1.9}}{ah_{1.9}+bh_{1.9}}$$

$$I_{Nav1.9}= Imax_{Nav1.9}\cdot Nav1.9_m^2\cdot Nav1.9_h\cdot(V_m-V_{Na})$$

3. Voltage-gated Nav1.7 channel

$$\frac{dNav1.7_m}{dt}=\frac{-Nav1.7_m+Nav1.7_{mss}}{\tau_{m_{Nav1.7}}}$$

$$\frac{dNav1.7_h}{dt}=\frac{-Nav1.7_h+Nav1.7_{hss}}{\tau_{h_{Nav1.7}}}$$

$$am_{1.7}=\frac{15.5}{1+e^{((V_m-5)/-12.08)}}$$

$$bm_{1.7}=\frac{35.2}{1+e^{((V_m+72.7)/16.7)}}$$

$$ah_{1.7}=0.24\cdot e^{(-\frac{V_m+115}{46.33})}$$

$$bh_{1.7}=4.32\cdot(1+e^{(\frac{V_m-11.8}{-12})})$$

$$\tau_{m_{Nav1.7}} = \frac{1}{am_{1.7}+bm_{1.7}}$$

$$\tau_{h_{Nav1.7}} = \frac{1}{ah_{1.7}+bh_{1.7}}$$

$$Nav1.7_{mss} = \frac{1}{1+e^{\left(\frac{Vm_{Nav1.7}-V_m}{kact_{Nav1.7}}\right)}}$$

$$Nav1.7_{hss} = \frac{1}{1+e^{\left(\frac{V_m+Vh_{Nav1.7}}{kinact_{Nav1.7}}\right)}}$$

$$I_{Nav1.7} = Imax_{Nav1.7} \cdot Nav1.7_m^2 \cdot Nav1.7_h \cdot (V_m - V_{Na})$$

4. Mechanosensitive Piezo2 channel

$$\frac{dPiezo_m}{dt} = \frac{Piezo_m+Piezo_{mss}}{\tau act_{Piezo}}$$

$$\frac{dPiezo_h}{dt} = \frac{Piezo_h+Piezo_{hss}}{\tau inact_{Piezo}}$$

$$Piezo_{mss} = \frac{1}{1+e^{\left(\frac{Vm_{Piezo}-Mechforce}{kact_{Piezo}}\right)}}$$

$$Piezo_{hss} = 1 - \frac{1}{1+e^{\left(\frac{Vh_{Piezo}-Mechforce}{kinact_{Piezo}}\right)}}$$

$$I_{PiezoNa} = Imax_{Piezo} \cdot Piezo_m^4 \cdot Piezo_h^2 \cdot (V_m - V_{Na})$$

$$I_{PiezoCa} = Imax_{Piezo} \cdot Piezo_m^4 \cdot Piezo_h^2 \cdot (V_m - V_{Ca})$$

$$I_{Piezo} = I_{PiezoNa} + I_{PiezoCa}$$

5. Mechanosensitive TRPA1 channel

$$\frac{dTRPA1_m}{dt} = \frac{-TRPA1_m+TRPA1_{mss}}{\tau act_{TRPA1}}$$

$$\frac{dTRPA1_h}{dt} = \frac{-TRPA1_h+TRPA1_{hss}}{\tau inact_{TRPA1}}$$

$$TRPA1_{mss} = \frac{1}{1+e^{\left(\frac{Vm_{TRPA1}-Mechforce}{kact_{TRPA1}}\right)}}$$

$$TRPA1_{hss}=1-\frac{1}{1+e^{(\frac{Vh_{TRPA1}-Mechforce}{kinact_{TRPA1}})}}$$

$$I_{TRPA1}= Imax_{TRPA1}\cdot TRPA1_m^2\cdot TRPA1_h\cdot(V_m-V_{Na})$$

6. Mechanosensitive two-pore TREK-1 channel

$$\frac{dTREK_m}{dt}=\frac{-TREK_m+TREK_{mss}}{\tau act_{TREK}}$$

$$TREK_{mss}=\frac{1}{1+e^{(\frac{Vm_{TREK}-Mechforce}{kact_{TREK}})}}$$

$$I_{TREK1}= Imax_{TREK}\cdot TREK_m^2\cdot(V_m-V_K)$$

Mechforce = 0.7, 4, 10, 20, 40, or 100 mN

7. pH-mediated ASIC3 channel

$$\frac{dASIC3_m}{dt}=\frac{-ASIC3_m+ASIC3_{mss}}{\tau act_{ASIC3}}$$

$$\frac{dASIC3_h}{dt}=\frac{-ASIC3_h+ASIC3_{hss}}{\tau inact_{ASIC3}}$$

$$\tau inact_{ASIC3}=197.36\cdot pH^2 - 1738.9\cdot pH + 3968.1$$

$$ASIC3_{mss}=\frac{1}{1+e^{(\frac{Vm_{ASIC3}-pH}{kact_{ASIC3}})}}$$

$$ASIC3_{hss}=1-\frac{1}{1+e^{(\frac{Vh_{ASIC3}-pH}{kinact_{ASIC3}})}}$$

$$I_{ASIC3}= Imax_{ASIC3}\cdot ASIC3_m\cdot ASIC3_h\cdot(V_m-V_{Na})$$

pH = 7.5

8. Voltage-gated Kv7.2 channel

$$\frac{dKv7_n}{dt}=\frac{-Kv7_n+Kv7_{nss}}{\tau_{nKv7}}$$

$$an_{Kv7}=k1act_{Kv7}\cdot e^{(\frac{V_m+Vm_{Kv7}}{k2act_{Kv7}})}$$

$$bn_{Kv7}=k1inact_{Kv7}\cdot e^{-(\frac{V_m+Vm_{Kv7}}{k2inact_{Kv7}})}$$

$$\tau_{n_{Kv7}}=\frac{1}{an_{Kv7}+bn_{Kv7}}$$

$$Kv7_{nss}=\frac{1}{1+e^{(\frac{-V_m-Vm_{Kv7}}{\tau act_{Kv7}})}}$$

$$I_{Kv7.2}= Imax_{Kv7}\cdot Kv7_n^2\cdot(V_m-V_K)$$

9. Delayed-rectifier Kv1.1 K^+ channel

$$\frac{dKv1.1_n}{dt}=\frac{-Kv1.1_n+Kv1.1_{nss}}{\tau act_{Kv1.1}}$$

$$Kv1.1_{nss}=\frac{\delta_{Kv1.1}}{1+e^{(\frac{Vm_{Kv1.1}-V_m}{kact_{Kv1.1}})}}$$

$$I_{Kv1.1}= Imax_{Kv1.1}\cdot Kv1.1_n^2\cdot(V_m-V_K)$$

10. Voltage-gated A-type K^+ channel

$$\frac{dKa_n}{dt}=\frac{-Ka_n+Ka_{nss}}{\tau_{n_{Ka}}}$$

$$\frac{dKa_{hfast}}{dt}=\frac{-Ka_{hfast}+Ka_{hfastss}}{\tau_{hfast_{Ka}}}$$

$$\frac{dKa_{hslow}}{dt}=\frac{-Ka_{hslow}+Ka_{hslowss}}{\tau_{hslow_{Ka}}}$$

$$Ka_{nss}=\frac{1}{1+e^{(\frac{-V_m-40.8}{9.5})}}$$

$$Ka_{hfastss}=\frac{1}{1+e^{(\frac{V_m+74.2}{9.6})}}$$

$$\tau_{n_{Ka}}= 1.2 + 2.56\cdot e^{(-2\cdot\left(\frac{V_m+60}{45.768}\right)^2)}$$

$$\tau_{hfast_{Ka}}= 25.46 + 67.41\cdot e^{(-2\cdot\left(\frac{V_m+50}{21.95}\right)^2)}$$

$$\tau_{hslow_{Ka}}= 200 + 587.4\cdot e^{(-\left(\frac{V_m}{47.77}\right)^2)}$$

$I_{Ka}= Imax_{Ka}\cdot Ka_n\cdot(0.3Ka_{hfast}+0.7Ka_{hslow})\cdot(V_m-V_K)$

11. Large-conductance Ca^{2+}-activated K^+ channel

$$\frac{dBKCa_n}{dt}=\frac{-BKCa_n+BKCa_{nss}}{\tau_{nBKCa}}$$

$p_{Ca}= \log_{10}\cdot(Ca_i e^{-3})$

$kact_{BKCa}= (-43.4\cdot p_{Ca})-203$

$sf_{BKCa}= 33.88 \cdot e^{-(p_{Ca}+5.42)/1.85^2}$

$$BKCa_{nss}=\frac{1}{1+e^{(\frac{kact_{BKCa}-V_m}{sf_{BKCa}})}}$$

$\tau_{nBKCa}=5.55 \cdot e^{\frac{V_m}{42.91}}+0.75-(0.12\cdot V_m)$

$I_{BKCa}= Imax_{BKCa}\cdot BKCa_n^2\cdot(V_m-V_K)$

12. T-type voltage-gated Ca^{2+} channel

$$\frac{dCaT_m}{dt}=\frac{-CaT_m+CaT_{mss}}{\tau act_{CaT}}$$

$$\frac{dCaT_h}{dt}=\frac{-CaT_h+CaT_{hss}}{\tau inact_{CaT}}$$

$$CaT_{mss}=\frac{1}{1+e^{(\frac{V_m-Vm_{CaT}}{kact_{CaT}})}}$$

$$CaT_{hss}=1-\frac{1}{1+e^{(\frac{V_m-Vh_{CaT}}{kinact_{CaT}})}}$$

$I_{CaT}= Imax_{CaT}\cdot CaT_m^2\cdot CaT_h\cdot(V_m-V_{Ca})$

13. L-type voltage-gated Ca^{2+} channel

$$\frac{dCaL_m}{dt}=\frac{-CaL_m+CaL_{mss}}{\tau act_{CaL}}$$

$$\frac{dCaL_h}{dt}=\frac{-CaL_h+CaL_{hss}}{\tau inact_{CaL}}$$

$$CaL_{mss}=\frac{1}{1+e^{(\frac{Vm_{CaL}-V_m}{kact_{CaL}})}}$$

$$CaL_{hss}=1-\frac{1}{1+e^{(\frac{Vh_{CaL}-V_m}{kinact_{CaL}})}}$$

$$hCa_{CaL}=\frac{1}{1+(Ca_i/1e^{-6})^4}$$

$$I_{CaL}= Imax_{CaL}\cdot CaL_m\cdot CaL_h\cdot hCa_{CaL}\cdot(V_m-V_{Ca})$$

14. NaK pump

$$I_{NaK}= Imax_{NaK}\cdot\frac{K_o^2}{K_o^2+KK_{NaK}^2}\cdot\frac{Na_i^{nH_{Na}}}{Na_i^{nH_{Na}}+KNa_{NaK}^{nH_{Na}}}\cdot\frac{V_m+70}{V_m+180}$$

15. PMCA pump

$$I_{PMCA}= Imax_{PMCA}\cdot\frac{Ca_i}{Ca_i+KCa_{PMCA}}$$

16. Na^+-Ca^{2+} exchanger

$$kqa= e^{\frac{0.35\cdot V_m}{k_{NCX}}}$$

$$kb_{NCX}= e^{\frac{-0.65\cdot V_m}{k_{NCX}}}$$

$$I_{NCX}= Imax_{NCX}\cdot (kqa\cdot Na_i^3\cdot Ca_o)-\frac{kb_{NCX}\cdot Ca_i\cdot Na_o^3}{(kNa^3+Na_o^3)\cdot(kCa+Ca_o)\cdot(1+0.1kb_{NCX})}$$

17. Passive K^+ leak channel

$$I_{Kleak}= Imax_{Kleak}\cdot(V_m-(-45))$$

ER mechanisms

1. IP_3 receptor flux

$$\frac{dhIP_3}{dt}= kf_{IP3}\cdot(kb_{IP3}-(Ca_i+kb_{IP3})\cdot hIP_3)$$

$$I_{IP3R} = \mathrm{Imax}_{IP3R} \cdot \left(\frac{IP_3}{IP_3 + k_{IP3}}\right) \cdot \left(\frac{Ca_i}{Ca_i + kCa_{IP3}} \cdot hIP_3\right)^3 \cdot \left(1 - \frac{Ca_i}{Ca_{ER}}\right)$$

2. SERCA pump

$$I_{SERCA} = \mathrm{Imax}_{SERCA} \cdot \left(\frac{Ca_i^2}{Ca_i^2 + KCa_{SERCA}^2}\right)$$

3. ER leak current

$$I_{leakER} = 5 \times 10^{-7} \cdot \left(1 - \frac{Ca_i}{Ca_{ER}}\right)$$

$$I_{leakER} = \mathrm{Imax}_{ERleak} \cdot \left(1 - \frac{Ca_i}{Ca_{ER}}\right) \qquad \text{if } Ca_i > KTCa$$

4. Ryanodine receptor flux

$$I_{CICR} = \mathrm{Imax}_{CICR} \cdot \left(\frac{Ca_i}{Ca_i + KCa_{CICR}}\right) \cdot (Ca_{ER} - Ca_i) \qquad \text{if } Ca_i > KTCa$$

$$I_{CICR} = 0$$

5. Ca_i^{2+} buffering in cytosol and ER

$$\beta_{ER} = \frac{CSQN \cdot K_{CSQN}}{(K_{CSQN} + Ca_{ER})^2}$$

6. PKC activation and signaling

$$\frac{d[RG_s]}{dt} = k_{rG} \cdot \mathrm{epsilon}_G \cdot [RTG] - \left(k_{rG} + \frac{k_{pG} \cdot [PGE_2]}{(K_{1G} + [PGE_2])}\right) \cdot [RG_s] - k_{rG}[RPG_s]$$

$$\frac{d[RPG_s]}{dt} = [PGE_2] \cdot \left(\frac{k_{pG} \cdot [RG_s]}{(K_{1G} + [PGE_2])}\right) \cdot \left(\frac{k_{eG} \cdot [RPG_s]}{(K_{2G} + [PGE_2])}\right)$$

$$\rho_{rG} = \alpha_G \left(\frac{[PGE_2] \cdot [RG_s]}{\mathrm{epsilon}_G \cdot [RTG] \cdot (K_{1G} + [PGE_2])}\right)$$

$$\frac{d[G_{\alpha q}]}{dt} = k_a(\delta + \rho_{rG})([G_T] - [G_{\alpha q}]) - k_d[G_{\alpha q}]$$

$$r_{hG}=\alpha_G\left(\frac{[Ca^{2+}]_i}{[Ca^{2+}]_i+k_{cG}}\right)G$$

$$\frac{d[PIP_2]}{dt}= -(r_{hG}+r_{rG})[PIP_2]-r_{rG}\cdot\Upsilon_G[IP_3]+r_{rG}[PIP_{2T}]$$

$$\frac{d[IP_3]}{dt}= \frac{r_{hG}}{\Upsilon_G}[PIP_2]-k_{degG}[IP_3]$$

$$\frac{d[PLC_{inact}]}{dt}= -(k_{PLCinact})[PLC_{act}]-k_{PLCact}\cdot[PLC_{inact3}]\cdot[G_{\alpha q}]$$

$$\frac{d[PLC_{act}]}{dt}= k_{PLCact}\cdot[PLC_{inact3}]\cdot[G_{\alpha q}]-(k_{PLCinact})[PLC_{act}]$$

$$\frac{d[DAG]}{dt}= k_{hyd}\cdot[PIP_2]\cdot[PLC_{act}]-k_{deg}[DAG]-kact_{PKC}[DAG]\cdot[PKC_{inact}] + (k_{inactPKC})[PKC_{act}] + k_{off}[DAG\_PKC]$$

$$\frac{d[PKC_{inact}]}{dt}= -k_{actPKC}\cdot[PKC_{inact}]\cdot[DAG] + k_{inactPKC}\ [PKC_{act}] + k_{off}[DAG\_PKC]$$

$$\frac{d[DAG\_PKC]}{dt}= k_{dp}[PKC_{act}] - k_{off}[DAG\_PKC]$$

$$\frac{d[PKC_{act}]}{dt}= k_{actPKC}\cdot[PKC_{inact}]\cdot[DAG] - k_{inactPKC}\ [PKC_{act}]- k_{dp}[PKC_{act}]-k_{Epacact}[Epac_{act}]$$

7. PKA activation and signaling

$$GPCR_{act}=\alpha_G\left(\frac{PGE_2\cdot[GPCR_{tot}]}{PGE_2+k_{EPdiss}}\right)$$

$$\frac{d[G_{\alpha s}]}{dt}=k1_{G\alpha s}[GPCR_{act}]\cdot\left(\frac{[G_{\alpha\beta\gamma}]}{K_{EPdiss}+[G_{\alpha\beta\gamma}]}\right)-k2_{G\alpha s}[G_{\alpha s}]$$

$$[G\alpha s_{inact}]=[G\alpha s_{tot}]-[G_{\alpha s}]-[G_{\alpha\beta\gamma}]$$

$$\frac{d[G_{\alpha\beta\gamma}]}{dt}= -k1_{G\alpha s}[GPCR_{act}]\cdot\left(\frac{[G_{\alpha\beta\gamma}]}{K_{EPdiss}+[G_{\alpha\beta\gamma}]}\right)+ k3_{G\alpha s}\left[G_{\beta\gamma}\right][G_{\alpha s}] - k4_{G\alpha s}\left[G_{\alpha\beta\gamma}\right]$$

$$\frac{d[G_{\beta\gamma}]}{dt}=k1_{G\alpha s}[GPCR_{act}]\cdot\left(\frac{[G_{\alpha\beta\gamma}]}{K_{EPdiss}+[G_{\alpha\beta\gamma}]}\right)- k3_{G\alpha s}\left[G_{\beta\gamma}\right][G_{\alpha s}] + k4_{G\alpha s}\left[G_{\alpha\beta\gamma}\right]$$

$$[AC_{act}]=\frac{[AC_{tot}]\cdot[G_{\alpha s}]}{[G_{\alpha s}]+kAC_{diss}}$$

$$[AC_{inact}]=[AC_{tot}]-[AC_{act}]$$

$$\frac{d[cAMP]}{dt}=\left(\frac{k5_{G\alpha s}[AC_{act}]}{kAC_{\beta\gamma diss}+[G_{\beta\gamma}]}\right)+k6_{G\alpha s}[AC_{act}]-k_{degcAMP}[cAMP]$$

$$\frac{d[RC]}{dt}=-k_{fPKA}[RC]\cdot[cAMP]^2+k_{bPKA}[RC_{cAMP2}]$$

$$\frac{d[RC_{cAMP2}]}{dt}=k_{fPKA}[RC]\cdot[cAMP]^2-k_{bPKA}[RC_{cAMP2}]-k_{f9}[RC_{cAMP2}]\cdot[cAMP]^2+k_{b9}[RC_{cAMP4}]$$

$$\frac{d[RC_{cAMP4}]}{dt}=k_{f9}[RC_{cAMP2}]\cdot[cAMP]^2-k_{b9}[RC_{cAMP4}]-k_{f10}[RC_{cAMP4}]+k_{b10}[RC_{cAMP}]\cdot[PKA]^2$$

$$\frac{d[RC_{cAMP}]}{dt}=k_{f10}[RC_{cAMP4}]-k_{b10}[RC_{cAMP}]\cdot[PKA]^2$$

$$\frac{d[PKA]}{dt}=2k_{f10}[RC_{cAMP4}]-k_{b10}[RC_{cAMP}]\cdot[PKA]^2$$

$$\frac{d[Epac_{inact}]}{dt}=-kf_{Epac}[Epac_{inact}]\cdot[cAMP]+kb_{Epac}[Epac_{act}]$$

$$\frac{d[Epac_{act}]}{dt}=kf_{Epac}[Epac_{inact}]\cdot[cAMP]-kb_{Epac}[Epac_{act}]$$

8. Equations for sensitization of nociceptor

First, we used the two equations shown below to compute the magnitude of change (ΔV_m for Nav1.7, Nav1.8, and Kv1.1, and ΔMech for TRPA1) induced by PKC and PKA:

$$\Delta V_{mPKC} \text{ or } \Delta Mech_{PKC}=\frac{15}{1+e^{\frac{khalf_{PKC}-[PKC]}{kslope_{PKC}}}}$$

$$\Delta V_{mPKA} \text{ or } \Delta Mech_{PKA}=\frac{13}{1+e^{\frac{khalf_{PKA}-[PKA]}{kslope_{PKA}}}}$$

where [PKC] and [PKA] denote the instantaneous concentrations of PKC and PKA (zero in the absence of an inflammatory mediator), respectively, and $khalf_{PKC}$, $khalf_{PKA}$, $kslope_{PKC}$, and $kslope_{PKA}$ denote the phosphorylation and steepness factors for PKC and PKA (Nicol et al., 1997; Wu et al., 2012). Next, we computed the new values of the activation and inactivation thresholds for each of the four ion channels using the following equations:

$$\left.\begin{aligned} Vact_{new\_i} &= Vact_i-\Delta V_{mPKC}-\Delta V_{mPKA} \\ Vinact_{new\_i} &= Vinact_i+\Delta V_{mPKC}+\Delta V_{mPKA} \end{aligned}\right\} \text{ Inflammatory mediator > 0}$$

$$\left.\begin{aligned} Vact_{new\_i} &= Vact_i+\Delta V_{mPKC}+\Delta V_{mPKA} \\ Vinact_{new\_i} &= Vinact_i-\Delta V_{mPKC}-\Delta V_{mPKA} \end{aligned}\right\} \text{ Inflammatory mediator = 0}$$

where i denotes one of the four channels, i.e., TRPA1, Nav1.7, Nav1.8, and Kv1.1; $V\mathrm{act}_{\mathrm{new\_i}}$ and $V\mathrm{inact}_{\mathrm{new\_i}}$ denote the new values; and $V\mathrm{act}_{\mathrm{i}}$ and $V\mathrm{inact}_{\mathrm{i}}$ denote the nominal values, respectively, of the activation and inactivation thresholds for each of the four channels.

9. ODEs for change in the activation and inactivation variables of Nav1.7, Nav1.8, Kv1.1, and TRPA1

$$\frac{\mathrm{d}[V\mathrm{hact}_{\mathrm{TTXs}}]}{\mathrm{d}t}=\left(\frac{V\mathrm{act}_{\mathrm{new}}\text{-}V\mathrm{hact}_{\mathrm{TTXs}}}{\tau_{\mathrm{Navphos}}}\right)$$

$$\frac{\mathrm{d}[V\mathrm{hinact}_{\mathrm{TTXs}}]}{\mathrm{d}t}=\left(\frac{V\mathrm{inact}_{\mathrm{new}}\text{-}V\mathrm{hinact}_{\mathrm{TTXs}}}{\tau_{\mathrm{Navphos}}}\right)$$

$$\frac{\mathrm{d}[V\mathrm{hact}_{\mathrm{TTXr}}]}{\mathrm{d}t}=\left(\frac{V\mathrm{act}_{\mathrm{new}}\text{-}V\mathrm{hact}_{\mathrm{TTXr}}}{\tau_{\mathrm{Navphos}}}\right)$$

$$\frac{\mathrm{d}[V\mathrm{hinact}_{\mathrm{TTXr}}]}{\mathrm{d}t}=\left(\frac{V\mathrm{inact}_{\mathrm{new}}\text{-}V\mathrm{hinact}_{\mathrm{TTXr}}}{\tau_{\mathrm{Navphos}}}\right)$$

$$\frac{\mathrm{d}[V\mathrm{hact}_{\mathrm{Kv1.1}}]}{\mathrm{d}t}=\left(\frac{V\mathrm{act}_{\mathrm{new}}\text{-}V\mathrm{hact}_{\mathrm{Kv1.1}}}{\tau_{\mathrm{Kv1.1phos}}}\right)$$

$$\frac{\mathrm{d}[TRPA1_{aM}]}{\mathrm{d}t}=\left(\frac{V\mathrm{act}_{\mathrm{new}}\text{-}TRPA1_{aM}}{\tau_{\mathrm{TRPA1phos}}}\right)$$

Nernst potential calculations

$$V_{\mathrm{Na}}=\frac{\mathrm{R\cdot T}}{\mathrm{zNa\cdot F}}\cdot\log\left(\frac{\mathrm{Na_o}}{\mathrm{Na_i}}\right)$$

$$V_{\mathrm{K}}=\frac{\mathrm{R\cdot T}}{\mathrm{zK\cdot F}}\cdot\log\left(\frac{\mathrm{K_o}}{\mathrm{K_i}}\right)$$

$$V_{\mathrm{Ca}}=\frac{\mathrm{R\cdot T}}{\mathrm{zCa\cdot F}}\cdot\log\left(\frac{\mathrm{Ca_o}}{\mathrm{Ca_i}}\right)$$

Ionic balances

$$\frac{\mathrm{dCa_i}}{\mathrm{d}t}=\text{-}((I_{\mathrm{CaT}}+I_{\mathrm{CaL}}+I_{\mathrm{PMCA}}-2I_{\mathrm{NCX}}+I_{\mathrm{PiezoCa}})/(\mathrm{zCa\cdot F\cdot 0.7vol}))\text{-}I_{\mathrm{SERCA}}\text{-}I_{\mathrm{leakER}}\text{-}I_{\mathrm{IP3}}\text{-}I_{\mathrm{CICR}})/(1/(1+370))$$

$$\frac{\mathrm{dCa_{ER}}}{\mathrm{d}t}=I_{\mathrm{SERCA}}\text{-}I_{\mathrm{leakER}}\text{-}I_{\mathrm{IP3}}\text{-}I_{\mathrm{CICR}}/\beta_{ER}$$

$$\frac{\mathrm{d}Na_i}{\mathrm{d}t} = -(I_{Nav1.8}+I_{Nav1.9}+I_{Nav1.7}+I_{PiezoNa}+I_{TRPA1}+3I_{NaK}+3I_{NCX})/(zNa\cdot F\cdot vol)$$

$$\frac{\mathrm{d}K_i}{\mathrm{d}t} = -(I_{TREK1}+I_{Kv7.2}+I_{Kv1.1}+I_{BKCa}+I_{Ka}+I_{Kleak}-2I_{NaK})/(zK\cdot F\cdot vol)$$

References

Bennett, M.R., Farnell, L., and Gibson, W.G. (2005). A quantitative description of the contraction of blood vessels following the release of noradrenaline from sympathetic varicosities. *J Theor Biol* 234(1)**,** 107-122. doi: 10.1016/j.jtbi.2004.11.013.

Leander, R., and Friedman, A. (2014). Modulation of the cAMP response by Gαi and Gβγ: a computational study of G protein signaling in immune cells. *Bull Math Biol* 76(6)**,** 1352-1375. doi: 10.1007/s11538-014-9964-4.

Lindskog, M., Kim, M., Wikstrom, M.A., Blackwell, K.T., and Kotaleski, J.H. (2006). Transient calcium and dopamine increase PKA activity and DARPP-32 phosphorylation. *PLoS Comput Biol* 2(9)**,** e119. doi: 10.1371/journal.pcbi.0020119.

Mohan, K., Nosbisch, J.L., Elston, T.C., Bear, J.E., and Haugh, J.M. (2017). A reaction-diffusion model explains amplification of the PLC/PKC pathway in fibroblast chemotaxis. *Biophys J* 113(1)**,** 185-194. doi: 10.1016/j.bpj.2017.05.035.

Nagaraja, S., Queme, L.F., Hofmann, M.C., Tewari, S.G., Jankowski, M.P., and Reifman, J. (2021). *In silico* identification of key factors driving the response of muscle sensory neurons to noxious stimuli. *Front Neurosci* 15**,** 719735. doi: 10.3389/fnins.2021.719735.

Nagaraja, S., Tewari, S.T., and Reifman, J. (2023). Identification of key factors driving inflammation-induced sensitization of muscle sensory neurons. *Front Neurosci* 17**,** 1147437. doi: 10.3389/fnins.2023.1147437.

Nicol, G.D., Vasko, M.R., and Evans, A.R. (1997). Prostaglandins suppress an outward potassium current in embryonic rat sensory neurons. *J Neurophysiol* 77(1)**,** 167-176. doi: 10.1152/jn.1997.77.1.167.

Wu, D.F., Chandra, D., McMahon, T., Wang, D., Dadgar, J., Kharazia, V.N., et al. (2012). PKCε phosphorylation of the sodium channel Nav1.8 increases channel function and produces mechanical hyperalgesia in mice. *J Clin Invest* 122(4)**,** 1306-1315. doi: 10.1172/JCI61934.
